# Basement Membrane and Cell Integrity of Self-Tissues in Maintaining *Drosophila* Immunological Tolerance

**DOI:** 10.1371/journal.pgen.1004683

**Published:** 2014-10-16

**Authors:** Moon Jong Kim, Kwang-Min Choe

**Affiliations:** Department of Systems Biology, Yonsei University, Seodaemun-gu, Seoul, South Korea; Stanford University, United States of America

## Abstract

The mechanism underlying immune system recognition of different types of pathogens has been extensively studied over the past few decades; however, the mechanism by which healthy self-tissue evades an attack by its own immune system is less well-understood. Here, we established an autoimmune model of melanotic mass formation in *Drosophila* by genetically disrupting the basement membrane. We found that the basement membrane endows otherwise susceptible target tissues with self-tolerance that prevents autoimmunity, and further demonstrated that laminin is a key component for both structural maintenance and the self-tolerance checkpoint function of the basement membrane. Moreover, we found that cell integrity, as determined by cell-cell interaction and apicobasal polarity, functions as a second discrete checkpoint. Target tissues became vulnerable to blood cell encapsulation and subsequent melanization only after loss of both the basement membrane and cell integrity.

## Author Summary

Autoimmune diseases may be caused by failures in the immune system or by altered selfness in target tissues; however, which of these is more critical is controversial. To better understand such diseases, it is necessary to first define the molecular mechanisms that provide self-tolerance to healthy tissues. As a model system, we used *Drosophila* melanotic mass formation, in which blood cells encapsulate degenerating self-tissues. By manipulating basement-membrane components specifically in target tissues, not in blood cells, we could elicit autoimmune responses against the altered self-tissues. Moreover, we found that at least two different checkpoints for self-tolerance operate discretely in *Drosophila* tissues. This parallels mammalian immunity and provides etiological insight into certain autoimmune diseases in which structural abnormalities precede immune system pathology, such as Sjögren's syndrome and type I diabetes mellitus.

## Introduction

The discovery of Toll-like receptors and other categories of pattern recognition receptors has greatly enhanced our understanding of how the immune system recognizes different types of pathogens [1], [2]; however, it is less clear why the immune system often turns its arsenal toward self-tissues. In fact, the same receptors that were originally found to bind specific types of pathogens are often involved in autoimmune diseases, making this issue more puzzling [3]. To understand this process, it is imperative to molecularly define the notion of the “immunological self”.

Autoimmune-like responses are also observed in invertebrates. In *Drosophila*, degenerating internal tissues are subjected to hemocyte (insect blood cell) encapsulation, in which large, flat lamellocytes wrap up the target tissues in layers and melanize them via the phenoloxidase cascade. This process is called melanotic mass formation [4]–[6]. The same process occurs as part of the immunological defense against oversized pathogenic invaders, such as parasitoid wasp eggs, which are too large to be engulfed by the most abundant phagocytic hemocytes, the plasmatocytes [7], [8]. Currently, about 100 genes have been found to display melanotic masses upon mutation or overexpression (see FlyBase.org). These genes are seemingly unrelated, and the specific triggers of this autoimmune-like reaction are largely obscure.

More than 30 years ago, Rizki and Rizki reported that the basement membrane (BM) appeared to serve as a barrier against hemocyte attack of self-tissues in *Drosophila* [9]–[11]. Whereas a same-species implant with the intact BM remained in the host, implants that had been mechanically damaged or pre-treated with collagenase to disrupt the BM triggered lamellocyte encapsulation [11]. Moreover, undamaged implants from sibling species did not induce lamellocyte encapsulation, whereas undamaged implants from distantly related species did, suggesting that hemocytes may recognize the molecular architecture of the BM of its own species. This interesting study raises several important questions as to which molecular component of the BM is crucial for blocking melanotic mass formation of self-tissues, and whether the BM is the sole surface feature for self-tolerance. Furthermore, their experiments were carried out in a sensitized genetic background, *tu(1)Sz^ts^*, in which the hemocytes were marginally hyperactive at a permissive temperature, which made it unclear whether the mass formation is caused primarily by defects in immune cells or target tissues. These questions have never been probed with genetic tools, largely due to the essential nature of the genes encoding the BM components collagen IV and laminin [12]–[16]. More recently, BM disruption was shown to act as a signal to recruit hemocytes to wound regions or to metastasizing tumors, providing further evidence for the BM-hemocyte relationship [17]–[19].

The BM is located on the basal side of epithelial tissues and serves multiple functions as a cell-supporting matrix, a tissue barrier, and ligands for cell surface receptors [20], [21]. The composition of the BM varies between tissue types, but in general, the BM contains the following four major components: collagen IV and laminin, which together form a meshwork, and the proteoglycan Perlecan and Nidogen, which function in the scaffold. The BM is maintained by evolutionarily conserved cell surface receptors, such as integrin and dystroglycan [22]. *Drosophila melanogaster* has two collagen IV genes, *Cg25C* (for α1 chain) and *vkg* (α2), and four laminin genes, *wb* (for laminin α1,2), *LanA* (α3,5), *LanB1* (β), and *LanB2* (γ) [16], [23]–[25]. Collagen IV is thought to exist mostly as Cg25C/Vkg heterotrimers, and LanB1 and LanB2 form the common core of the two laminin trimers, laminin W and laminin A. Thus, the mutant phenotypes of these genes are very similar in their own categories, and absence of one subunit is known to prevent BM incorporation of the other(s) [14], [15], [26]. The major BM components are expressed and secreted predominantly by the fat body and hemocytes [12], [26], [27], although laminins are also expressed in various other tissues [16], [27].

Here, we genetically removed each of the major BM components using RNA interference (RNAi) followed by careful immunohistochemical analysis and examined their roles in melanotic mass formation. We discovered that lamellocyte encapsulation may be blocked by two separate and discrete self-tolerance checkpoints that operate in healthy target tissues. The first checkpoint involves laminin of the BM, and the second involves cell integrity as determined by cell-cell adhesion and apicobasal cell polarity.

## Results

### BM disruption induces melanotic mass formation

**Figure 1:**
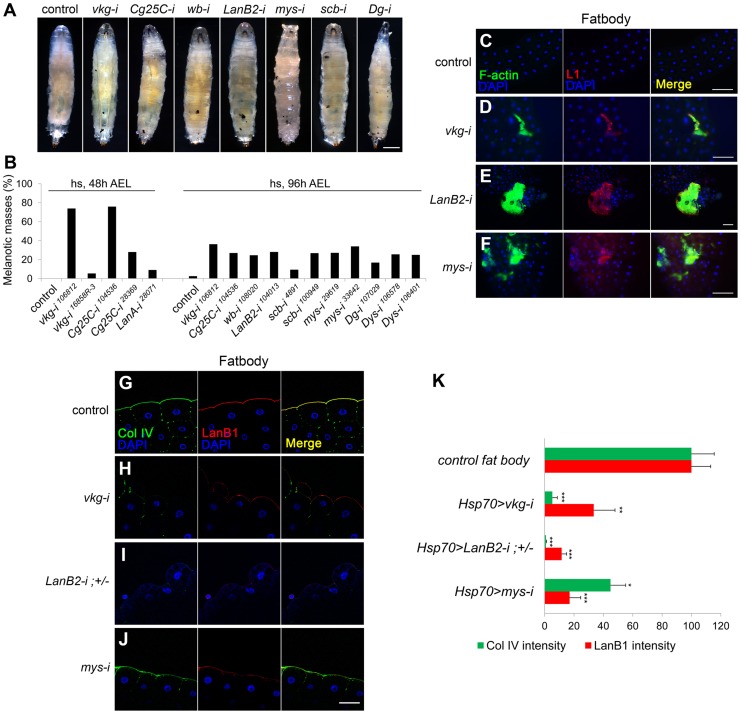
BM disruption induces melanotic mass formation. (A, B) Pictures (A) and percentages (B) of the third instar larvae of the different genotypes containing melanotic nodules. *Hsp70-GAL4* was used. The control represents *GAL4* only. n>150 for each genotype. Heat shock was carried out as described in Methods. (C–F) The larval fat bodies were analyzed for lamellocyte encapsulation. *Hsp70>GAL4* was used. The control was *GAL4* only. Anti-L1 (red) and phalloidin-FITC (green) marked F-actin-rich lamellocytes. Nuclei were stained with DAPI (blue). (G–J) Confocal images of the fat body BM. *Hsp70-GAL4* was used. The control was *GAL4*-only. *LanB2-i;+/−* indicates *Hsp70>LanB2-i;LanB2^+^/LanB2^−^*. Collagen IV (Col IV), laminin, and nuclei were visualized after staining with anti-Col IV (green), anti-LanB1 (red), and DAPI (blue), respectively. (K) Quantitation of the fluorescence intensities for collagen IV in the BM after staining with anti-Col IV antibody (green) and for BM laminin after staining with anti-LanB1 (red). Error bars represent standard errors of the mean (SEM). *p<0.05, **p<0.01, and ***p<0.001 by Student's *t* test. For each genotype, n≥5. Scale bar: 500 µm (A), 100 µm (C–F), and 50 µm (G–J).

To systematically investigate the relationship between the BM and the melanotic mass phenotype, we disrupted the BM using genetic approaches. We knocked down genes for the two collagen IV subunits and the four laminin subunits individually via *UAS-RNAi* using ubiquitous (*Act5C-GAL4*), inducible (*Hsp70-GAL4*), and tissue-specific *GAL4* drivers (*HmlΔ-GAL4*, *FB-GAL4*, and *Cg-GAL4*) (summarized in Table S1). Knockdown of any one of the six genes consistently induced black masses in the larvae with either *Hsp70-GAL4* or *Cg-GAL4* drivers (Figure 1A and 1B; Table S1). Knockdown of the genes for the BM receptor integrins (*scb* for αPS3 and *mys* for βPS), Dystroglycan (*Dg*), or its cytosolic adaptor Dystrophin (*Dys*) similarly induced black masses (Figure 1A and 1B; Table S1). Melanotic masses formed mainly in fat bodies and salivary glands. We analyzed the fat bodies of these larvae by immunostaining with the lamellocyte-specific L1 antibody. Pale brown-colored fat bodies (dissected in early stages of melanin deposition) from larvae in which collagen IV, laminin, or integrin had been knocked down by RNAi were encapsulated by a few lamellocytes (Figure 1C–F). Black nodules (dissected in late stages of melanin deposition) recovered from these larvae were also L1-positive (Figure S1A). Using confocal microscopy, we confirmed the complete disappearance or severe disruption of the BM in the fat bodies of these larvae (Figure 1G–K). This immune response did not appear to be caused by pathogen infection, as the larvae did not induce the antimicrobial peptide gene *Attacin-A* (Figure S1B; Text S1). Thus, these data indicate that BM loss induced melanotic mass formation.

### Analysis of the BM in extant melanotic mass mutants

**Figure 2:**
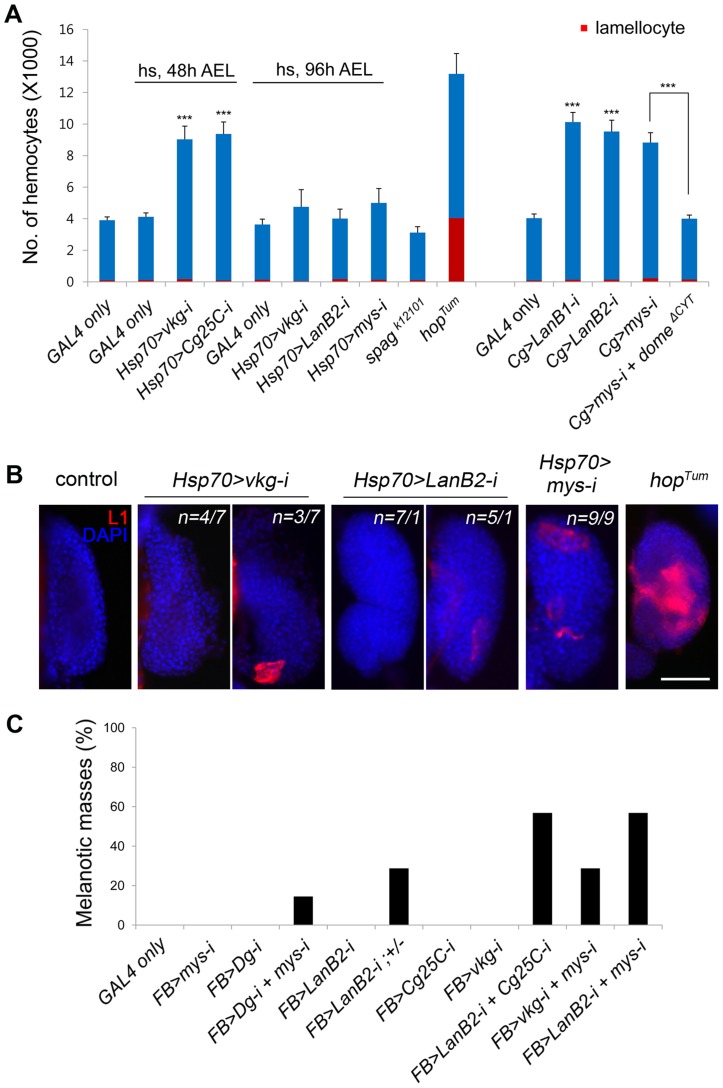
Melanotic mass formation in the BM-deficient larvae is a normal immune response against altered self. (A) Numbers of circulating hemocytes (blue+red) and lamellocytes (red) in each larva were counted in the various genotypes. *Cg-GAL4* drives gene expression in hemocytes and the fat body. *hop^Tum^* was raised at 29°C. Error bars represent SEM. ***p<0.001 by Student's *t*-test. Percentages of larvae containing melanotic masses: *Cg>mys-i*, 27.38% (n = 141/515); *Cg>mys-i dome^ΔCYT^*, 32.00% (n = 72/225). (B) Lamellocyte differentiation in the lymph gland was analyzed by staining with anti-L1 antibodies (red) in the larvae of the indicated genotypes. The nuclei were stained with DAPI (blue). For the *vkg* and *LanB2* knockdowns, two classes were observed, the frequency of which plus an example is shown. The control panel represents Oregon R. *hop^Tum^* was raised at 25°C. Scale bar: 50 µm. (C) Melanotic mass formation after knockdown of genes for collagen IV, laminin, and integrin in various combinations using the fat body-specific *FB-GAL4*. For each genotype, n>150.

To determine whether loss of the BM is a general feature of the melanotic mass phenotype, we examined various genes that had been previously associated with the melanotic mass using mutant or RNAi-treated larvae. Because we were primarily interested in the target tissues as opposed to hemocytes, we first excluded mutants that might be classifiable as “true blood cell tumors”, in which melanotic mass formation was due to hemocyte hyperactivation [4]. The following four genes were analyzed for the BM: *spag* [6], *krz* [6], *mRpS30* [28], and *hyx* [28]. We found that mutant or RNAi-treated larvae for these genes commonly had disrupted BMs in the fat bodies and that the fat bodies were positive for L1 (Figure S1C and S1D). We also examined 30 other melanotic mass-associated genes; however, the RNAi-treated larvae did not reproduce black nodules with the available *UAS* transgenes and tissue-specific *GAL4* drivers or exhibited early lethality with either ubiquitous or stronger *GAL4* drivers, thus precluding further analysis (Table S2). We also analyzed *hop^Tum^* larvae, in which the JAK kinase Hopscotch is constitutively active and melanotic mass phenotype is dominant at restrictive temperatures (>25°C) [29]. Although the melanotic phenotype of this mutant may fit the classification for the blood cell tumors [30] in that hemocyte numbers increase dramatically (see Figure 2A and 2B), we sought to determine its target tissues. At 25°C, only the collagen IV level decreased severely, while at the restrictive temperature (29°C), both collagen IV and laminin were absent, and numerous lamellocytes attached to the fat body and the salivary gland (Figure S1C and S1D). Altogether, these observations corroborated the evidence that BM-deficient tissues induce melanotic masses.

### Melanotic mass formation in BM-deficient larvae is an autoimmune response against altered self

To see whether lamellocyte encapsulation of BM-deficient tissues was a normal hemocyte reaction to abnormal self-tissue or rather due to an abnormality in the hemocyte itself [4], we assessed the activation state of hemocytes first by counting the cells. The numbers of circulating hemocytes of some of the BM-deficient larvae were 2–2.5-fold higher than those of controls (Figure 2A); however, the numbers of lamellocytes and crystal cells, a third type of hemocytes that contain phenol oxidation enzymes as a crystal form in their cytosol [31], did not increase significantly in any of the cases (Figure 2A, S2A, and S2B; Text S1). This result was in stark contrast to the results for *hop^Tum^* (Figure 2A), *Toll^D^*, or *cactus* mutants, which harbor hyperactive hemocytes [5], [30]. To inhibit hemocyte hyperproliferation displayed by some of the BM-deficient larvae, we expressed a dominant-negative allele for the JAK/STAT pathway receptor Domeless (*dome^ΔCYT^*) in *mys* RNAi larvae [18], [32]. Hemocyte numbers were restored to a normal level in these larvae; however, melanotic mass formation was not abrogated or reduced, indicating that the mass phenotype was not due to hemocyte hyperproliferation (Figure 2A).

We then examined the larval hematopoietic lymph gland, as robust lamellocyte differentiation in the lymph gland is a common feature of blood cell tumors [28], [33]–[36]. The lymph glands of wild-type larvae rarely contained the L1-positive lamellocytes [37] (Figure 2B). The lymph glands of collagen IV- or laminin-knockdown larvae occasionally contained 1–5 L1-positive cells, while lymph glands of integrin-knockdown larvae had 5–20 of these cells. The observed levels of activation may be expected for melanotic mass-forming larvae, but the levels were significantly different from that of *hop^Tum^* in which the cortical zone of the lymph gland was filled with L1-positive cells [38] (Figure 2B). We then examined sessile hemocytes, another source of lamellocytes upon wasp egg infection [39]. Collagen IV knockdown did not change the sessile hemocyte population, as analyzed by plasmatocyte-specific *Eater-GFP* (Figure S2C). Finally, we knocked down the BM genes using the fat body-specific *FB-GAL4* [28] to determine whether gene manipulation at the target site only, and not in the hemocyte or in the hematopoietic organs, still induced melanotic masses. Knockdown using any of the available *UAS-RNAi* transgenes singly did not induce melanotic masses, presumably due low knockdown efficiencies with *FB-GAL4* driver (Figure 2C); however, knockdown of various combinations of the transgenes induced melanotic masses specifically in the fat body in the absence of a sensitized genetic background [11]. We obtained similar results following fat body-specific RNAi knockdown for integrins or Dystroglycan, which should act strictly in a cell-autonomous manner (Figure 2C). Circulating hemocytes produced collagen IV and laminin, as reported previously [12], [26], [27], but were not enclosed by a sheet of collagen IV or laminin outside of the cell (Figure S2D; Text S1), excluding the possibility that knockdown of these components in the fat body may have produced the mass phenotype by affecting the surface of the hemocyte rather than the target. Based on these results, we conclude that melanotic mass formation in BM-deficient larvae results from a normal immune response (operationally defined by *FB-GAL4*) against altered self and is not ascribed to a failure in the immune system.

### Laminin deficiency in the BM induces melanotic masses

**Figure 3:**
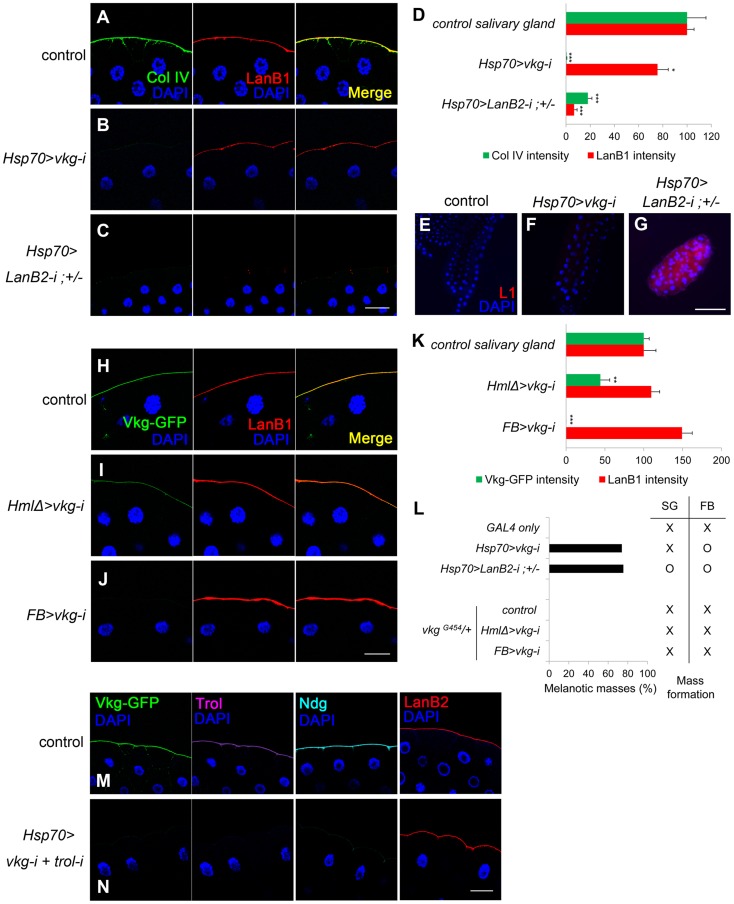
Laminin alone sufficiently blocks melanotic mass formation. (A–C) Confocal images of salivary-gland BMs after staining for collagen IV (anti-Col IV in green), laminin (anti-LanB1 in red), and nuclei (DAPI in blue). The control is *GAL4*-only. (D) Quantitation of the fluorescence intensities in (A–C). Error bars represent SEM. *p<0.05, ***p<0.001 by Student's *t*-test. (E–G) Salivary glands from (A–C) after staining lamellocytes using anti-L1 antibodies (red) and the nuclei with DAPI (blue). (H–J) Confocal images of salivary-gland BMs after visualizing collagen IV using Vkg-GFP (green), laminin using anti-LanB1 antibodies (red), and nuclei using DAPI (blue). *HmlΔ-GAL4* and *FB-GAL4* drive gene expression in hemocytes the fat body, respectively. The control represents *vkg^G454^*/+. (K) Quantitation of the fluorescence intensities in (H–J). Error bars represent SEM. **p<0.01 and ***p<0.001 by Student's *t*-test. (L) Percentages of larvae containing melanotic masses in (A–C) and (H–J) and their mass-forming positions. SG and FB indicate salivary gland and fat body, respectively. (M, N) Confocal images of the salivary-gland BMs of *vkg^G454^*/+ (M) and *vkg^G454^ UAS-vkg-i/+ +; Hsp70-GAL4/UAS-trol-i* (N) larvae after visualization of collagen IV using Vkg-GFP (green), Perlecan (Trol) using anti-Trol antibodies (purple), Nidogen using anti-Ndg antibodies (cyan), laminin using anti-LanB2 antibodies (red), and nuclei using DAPI (blue). Scale bar: 50 µm (A–C, H–J, M, N) and 200 µm (E–G).

To investigate which component of the BM is crucial in self-tolerance, we individually knocked down each of the BM genes using various *GAL4* drivers and analyzed BM integrity and the melanotic mass phenotype. Since melanotic masses in the BM-deficient larvae formed mainly in fat bodies and salivary glands, we focused on these two organs. In fat bodies, collagen IV knockdown with *Hsp70-GAL4* reduced not only collagen IV (the fluorescence intensity was 5.23% of the control level) but also laminin in the BM (hereafter, BM laminin) effectively (33.46%; Figure 1H and 1K). Similarly, laminin knockdown (11.56%) nearly completely eliminated the BM collagen IV (0.91%; Figure 1I and 1K), indicating that these factors are structurally inter-dependent in this organ. In salivary glands, however, collagen IV knockdown with the same *Hsp70-GAL4* eliminated only collagen IV (0.47%), while leaving 75.61% of laminin in the BM (Figure 3B and 3D), allowing for separation of the two components in this organ. Laminin knockdown (6.71%) effectively removed the BM collagen IV (18.02%) in the salivary gland (Figure 3C and 3D), as in the fat bodies. These results were consistent with the fact that laminin is the key component of BM assembly [14], [20], [21]. More importantly, collagen IV knockdown induced melanotic masses only in the fat body and not in the salivary gland, whereas laminin knockdown induced melanotic masses in both organs (Figure 3E–G and L, the first four experiments; melanotic encapsulation often occurred regionally but not in the entire organs, which might be due to incomplete removal of the BM laminin). These results strongly suggest that BM laminin and possibly other factors that are tightly associated with laminin block melanotic mass formation, whereas collagen IV is not necessary for blocking this process as the collagen IV-deficient larvae did not form melanotic masses in the salivary gland (Figure 3F and 3L).

### Laminin is the key element in the self-tolerance checkpoint

To further explore the self-tolerance checkpoint function of the BM components, we knocked down the collagen IV and laminin genes using *HmlΔ-GAL4* and *FB-GAL4*, which are active in hemocytes and fat bodies, respectively [28], [40]. Laminin knockdown using these *GAL4* drivers was inefficient. As for collagen IV knockdown, the changes in BM integrity induced by these two *GAL4* drivers were generally the same as those with *Hsp70-GAL4* (Figure 3H–K), except that collagen IV knockdown specifically eliminated the BM collagen IV but left considerable laminin at the BM even in the fat bodies (Figure S3A–D). These larvae did not form black masses, further demonstrating that collagen IV is dispensable in the BM for blocking melanotic masses (Figure 3L, the last four experiments).

We next examined Perlecan and Nidogen, the other two major components of the BM. Knockdown of the Perlecan-coding gene *trol* with *Act5C-GAL4* did not induce black masses, indicating that Perlecan is not required in this process (Figure S3E; Table S1). Knockdown of Nidogen using available RNAi transgenes were unsuccessful; however, we found that Nidogen was completely absent in collagen IV-deficient, melanotic mass-free larvae, indicating that Nidogen is not required for blocking melanotic mass formation (Figure S3F; Table S1). Finally, we knocked down both *trol* and *vkg*, leaving laminin as the only major BM component, and found that melanotic masses were not formed (Figure 3M and 3N). Thus, these results indicate that the BM laminin was sufficient to block melanotic mass formation against self-tissue.

### Loss of cell integrity correlates with melanotic mass formation

**Figure 4:**
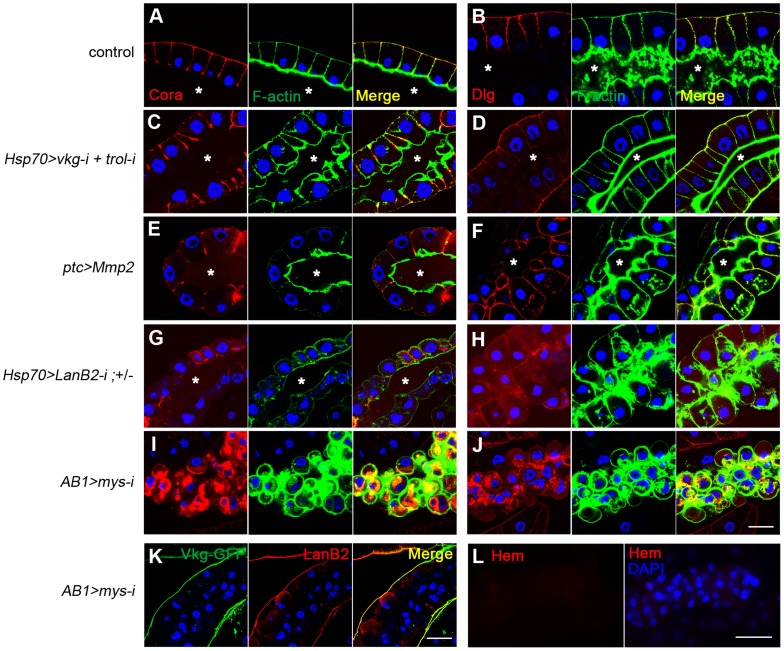
Cell integrity is severely disrupted specifically in BM-deficient, melanotic mass-containing larvae. (A–J) Confocal images of larval salivary glands from the indicated genotypes illustrate defects in apicobasal cell polarity and cell-cell adhesion. Cora and Dlg were stained with anti-Cora (red; A, C, E, G, I) and anti-Dlg (red; B, D, F, H, J), respectively. F-actin and the nuclei were stained with phalloidin-FITC (green) and DAPI (blue), respectively. *ptc-GAL4* is active in the salivary gland and various other tissues. *AB1-GAL4* is active in the salivary gland. The control was Oregon R. Asterisks mark the lumen. (K) Confocal images of the salivary-gland BMs of *AB1>mys-i* larvae showing BM collagen IV (Vkg-GFP in green) and BM laminin (anti-LanB2 in red). (L) Confocal images of the salivary gland revealed that the organ surface is negative for the pan-hemocyte marker Hemese (Hem, red). Nuclei were stained with DAPI (blue). Scale bar: 50 µm (A–J, K) and 100 µm (L).

As an independent approach to determine BM function, we removed the BM by overexpressing Matrix metalloproteinase 2 (Mmp2). Mmp2 expression in the salivary gland severely disrupted BM integrity, as reported previously [18] (Figure S4A and S4B), but contrary to our expectations, the larvae did not form melanotic masses. Hemocytes attached to the salivary gland, indicating that hemocyte access to the target cells was not blocked (Figure S4C and S4D). We noticed that the salivary gland cells of these larvae looked similar to those of wild-type larvae (Figure 4A and 4B vs. 4E and 4F), whereas the cells of laminin-knockdown larvae were dissociative and round (Figure 4G and 4H), indicative of the loss of cell-cell adhesion. We further examined whether these melanotic mass-containing larvae had defects in cell polarity using apicobasal cell polarity markers [41]. In wild-type larvae, Cora and Dlg localized to the lateral and basal sides of cells (Figure 4A and 4B). A similar pattern was observed in *vkg trol* RNAi larvae (Figure 4C and 4D). In *ptc>Mmp2* larvae in which Mmp2 is expressed in the salivary gland [18], the lumen often twisted as it expanded, and cell arrangement occasionally became abnormal (Figure 4F). Dlg diffused throughout the membrane, albeit weakly (Figure 4F). Nevertheless, Cora was still excluded from the apical side (Figure 4E), indicating that apicobasal polarity was partially maintained, perhaps due to the remains of the disrupted BM on the cell surface (Figure S4B). In contrast, the salivary gland cells of laminin-knockdown larvae displayed complete loss of apicobasal polarity. Cora and Dlg were localized throughout the cell membrane and were more often lost from the membrane in these larvae (Figure 4G and 4H). Cell-cell contacts were also severely disrupted. These results strongly suggest that loss of cell integrity, in addition to the loss of the BM, may be required for melanotic mass formation. In this report, we will subsequently use the term “cell integrity” to refer to the cellular aspects involving cell-cell adhesion and cell polarity.

### Cell integrity and the BM are two discrete checkpoints for self-tolerance

**Figure 5:**
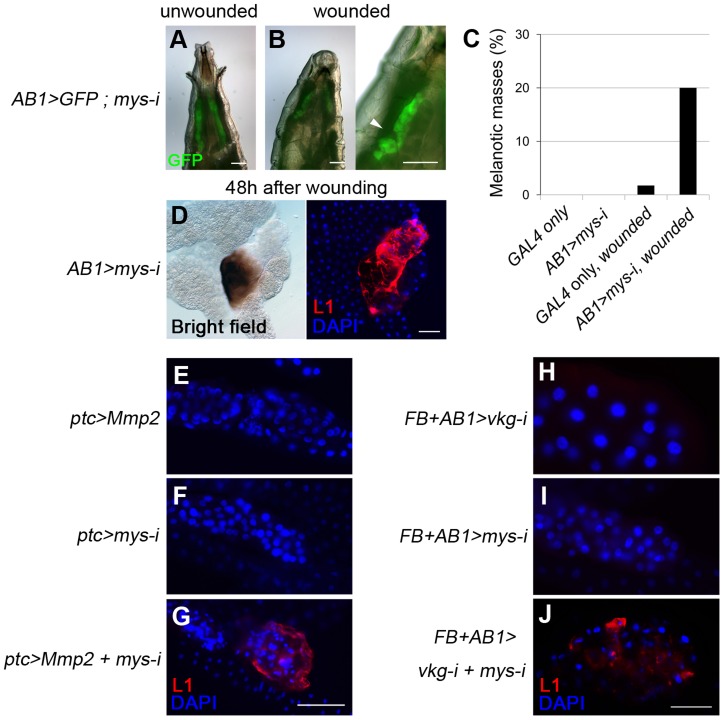
Loss of cell integrity is required in addition to BM disruption for melanotic mass formation. (A, B) Mechanical disruption (arrowhead) of the salivary-gland BM of *AB1>mys-i, GFP* larvae with GFP expression (green) in the salivary gland. (C) Melanotic mass formation in pinch-wounded larvae of *AB1>GFP* control (n = 1/57) and *AB1>mys-i, GFP* (n = 17/84) larvae. (D) Melanized salivary glands of wounded *AB1>mys-i* larvae were positive for L1 (red). Nuclei were stained with DAPI (blue). (E–J) Confocal images of the larval salivary glands of the indicated genotypes after staining lamellocytes with anti-L1 antibodies (red) and nuclei with DAPI (blue). Scale bar: 200 µm (A, B, E–G) and 100 µm (D, H–J).

To examine the possible role of cell integrity as another checkpoint, we used melanotic mass-free *AB1>mys-i* larvae. Integrin knockdown with the salivary gland driver *AB1-GAL4* [42] did not disrupt the BM but resulted in detachment of the BM from the salivary gland tissue (Figure 4K). The cells lost both cell polarity and adhesion properties, and as a result, the BM appeared as a sack containing sticky balls (Figure 4I–K). As expected, hemocytes were not detected on the surface of the salivary gland in these larvae (Figure 4L). To explore this phenotype in more depth, we first mechanically sheared the salivary gland BM by pinching the *AB1>mys-i*, *GFP* larva at its anterior side with forceps (Figure 5A and 5B). After two days, these larvae developed black masses in the salivary gland at a rate that was 12-fold higher than that of the pinched control larvae (Figure 5C). Melanized salivary glands dissected from the wounded larvae were positive for L1 (Figure 5D). Second, we enzymatically disrupted the salivary gland BM of *ptc>mys-i* larvae by overexpressing Mmp2, as a means to more specifically manipulate the larvae. These larvae developed black masses, and the salivary glands dissected from the larvae were positive for L1 (Figure 5E–G). In these experiments, *ptc-GAL4* and *mys-i^27735^* were used instead of the previously used *AB1-GAL4* and *mys-i^33642^* because the latter combination with *UAS-Mmp2* caused severe growth retardation of salivary glands. The reproducibility of the knockdown phenotypes was confirmed using *mys-i^27735^* (Figure S5). Third, we tried to wear out the BM by reducing levels of collagen IV in *mys*-i larvae. Black masses formed only in the fat bodies of *FB>vkg-i*, *mys-i* larvae (Figure 2C): but due to the additional *AB1-GAL4* driver, a few of *FB+AB1>vkg-i*, *mys-i* larvae developed black masses also in the salivary glands, and again, the salivary glands of those larvae were positive for L1 (Figure 5J). Neither *mys*-i nor *vkg*-*i* alone formed black masses in the salivary gland with the same *FB+AB1 GAL4* drivers (Figure 5H and 5I). Taken together, our data demonstrate that cell integrity is an additional and discrete checkpoint for tolerance to self-tissues. We sought to define cell integrity in this system by knocking down genes known to be involved in cell-cell adhesion and cell polarity. Knockdown of either *scrib*, *dlg*, *cora*, *FasIII*, *shg*, or *arm* together with Mmp2 overexpression, however, did not induce melanotic mass formation, indicating that loss of any of these components at least singly did not affect cells sufficiently for disrupting the cell-integrity checkpoint function.

## Discussion

**Figure 6:**
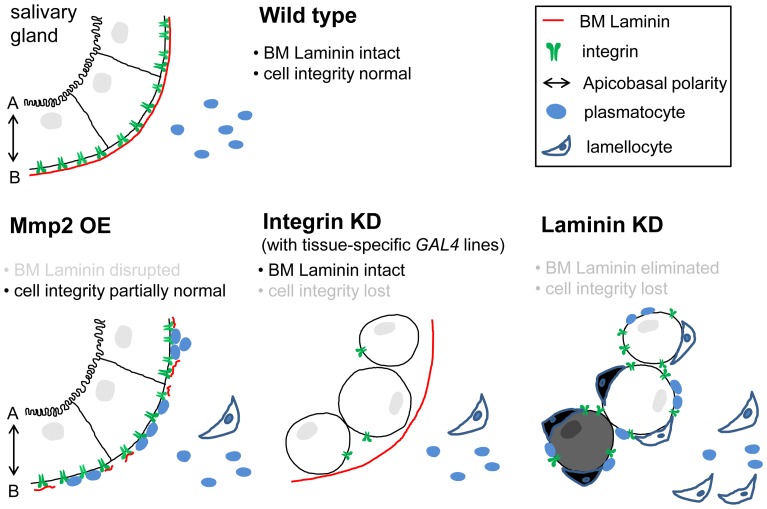
Proposed model for the self-tolerance checkpoint function in the *Drosophila* immune system. Epithelial tissues are equipped with at least two self-tolerance checkpoints: BM laminin and cell integrity. The latter is currently less well-defined but appears to include apicobasal cell polarity and cell-cell adhesion. With either one of the checkpoints, self-tissue may become tolerant to the immune system of its own. Upon Mmp*2* overexpression (OE), the salivary gland loses BM integrity to an extent that allows plasmatocyte access; however, cell integrity remains largely intact (intact 2^nd^ checkpoint). Upon integrin knockdown (KD) in the salivary gland (*AB1>mys-i*), cells lose cell integrity, while the organ maintains the BM (intact 1^st^ checkpoint). In the presence of laminin knockdown, both of the self-tolerance checkpoints are non-functional, and the tissues are subjected to lamellocyte encapsulation. Avirulent parasitoid eggs or wing disc implants from distantly related species [11] do not have either of the checkpoints that are compatible with the host immune system, and thus, these foreign bodies are also sequestered by lamellocyte encapsulation.

Based on the results of these studies, we propose that BM laminin on target tissues functions as a crucial component not only in BM assembly but as a tolerance checkpoint to self-tissues (Figure 6). As a self-tolerance checkpoint, the BM may either serve as a physical barrier or provide an inhibitory ligand for hemocyte receptors. We speculate that the latter is the case for several reasons. First, the heterospecific implantation experiments described above suggest that the hemocyte recognizes the BM structure of its own species [11]. Second, insect hemocytes are known to encapsulate a wide range of foreign materials, from parasitoid wasp eggs to synthetic beads, when injected into the hemocoel [37], [43], [44]. Encapsulation of parasites is faster than encapsulation of non-parasitic, heterospecific implants [discussed in 11]. Thus, hemocytes must be equipped with various cell surface receptors, including some as activating receptors with different binding spectra for pathogen-associated molecular patterns, and others as inhibitory receptors with narrow binding specificities to self-tissues. More specifically, laminin-coating of Sephadex beads inhibit melanotic encapsulation of the beads in mosquito hemocoel [43]. The outer surface of the *Plasmodium* oocyst appears to bind to mosquito-derived laminin upon passage through the midgut epithelium of the mosquito [45], suggesting that the insect laminin may indeed serve as an inhibitory ligand to hemocytes of its own species.

Furthermore, we have identified cell integrity as another checkpoint for self-tolerance. Self-tissues must lose both the BM and cell integrity in order to be subjected to lamellocyte encapsulation (Figure 6). The two checkpoints are indeed discrete and experimentally separable. In our experimental system, BM integrity or cell integrity could be disrupted in the salivary gland selectively via *ptc>Mmp2* or *AB1>mys-i*, respectively. The salivary glands of these larvae are subjected to melanotic encapsulation if the tissues fail to acquire the state of self-tolerance from the other, remaining checkpoint. Therefore, multiple failures on sequential self-tolerance checkpoints elicit autoimmune responses, which is analogous to the mechanism of self-tolerance in the mammalian adaptive immune system [46]. In this respect, BM laminin is unique in that knockdown of laminin disrupted both BM integrity and cell integrity simultaneously. During pharyngeal tube formation of the *C. elegans* embryo, laminin is required for establishment of cell polarity of a group of precursor cells called the double plate [47]. Since this process occurs before the tissue BM is formed and the mutant phenotype is not shared by collagen IV or perlecan mutants, the authors concluded that this function of laminin is distinguished from its role in the BM. Our results indicate that the *Drosophila* laminin functions similarly and thus is unique among the BM components.

Since cell integrity was identified as a discrete self-tolerance checkpoint, it is interesting that target tissue is not subjected to encapsulation during wound healing or developmental processes in which BM integrity is disrupted temporarily. Upon tissue damage, circulating plasmatocytes attach to the wound region as early as 5 min [17], presumably to help remodel the tissue, including the disrupted BM, as hemocytes also produce BM components [12], [27] (Figure 3I). According to our model, as long as cell integrity remains intact, this checkpoint would warrant self-tolerance and block lamellocyte encapsulation in these cases, and thus, wound repair would proceed safely (as in Figure 5C). Cancer metastasis is a more complex problem, as the hallmarks of cancer include BM degradation and loss of epithelial polarity [48], [49]. Additional work will be required to probe this situation, but it is plausible that cell integrity may exert its checkpoint function normally through inhibitory cytokines, and prior to successful metastasis, cancer cells may have to obtain the ability to secret such cytokines independently of the state of cell integrity.

While our results were obtained in the invertebrate *Drosophila* system, we think these findings have relevance to mammalian autoimmunity. In type I diabetes in humans and in a mouse model, leukocyte infiltration and subsequent β cell destruction have been shown to correlate with disruption of the peri-islet BM, suggesting that the BM may protect islets from autoimmune attack [50], [51]. In Sjögren's syndrome, which mainly affects the exocrine tissues such as tear and salivary glands, increased degradation of the basal lamina is observed in labial salivary glands, and changes in laminin composition of the salivary acini correlates well with disease progression [52], [53]. A clear causal relationship is still lacking, but it is tempting to speculate that at least some of the autoimmune diseases may be caused by alterations in BM integrity and/or cell polarity and loss of these features may be recognized by the immune system as “missing self”, similar to the way natural killer cells distinguish between self and nonself [54], [55].

## Methods

### Fly strains

The following strains were obtained from public stock centers: *UAS-vkg-i* (106812^†^), *UAS-Cg25C-i* (104536, 28369), *UAS-LanA-i* (18873), *UAS-wb-i* (108020), *UAS-LanB1-i* (23119, 23121), *UAS-LanB2-i* (42559, 42560, 104013^†^), *UAS-trol-i* (22642), *UAS-Ndg-i* (13208), *UAS-mew- i* (44890), *UAS-if-i* (100770, 44885), *UAS-scb-i* (4891, 100949), *UAS-ItgαPS4-i* (37172) *UAS- ItgαPS5-i* (6646, 100120), *UAS-mys-i* (29619^†^), *UAS-Itgβν-i* (893), *UAS-Dg-i* (107029), *UAS-Dys-i* (106401, 106578), and *UAS-krz-i* (103756^†^) from Vienna *Drosophila* RNAi Center; *UAS-vkg-i* (16858R-3), *UAS-trol-i* (12497R-1), *UAS-Ndg-i* (12908R-3), *UAS-mew- i* (1771R-1), *UAS- ItgαPS4-i* (16827R-2), *UAS- Itgβν-i* (1762R-1), *UAS-mys-i* (1560R-1), *UAS-mRpS30-i* (8470R-4^†^), *UAS-hyx-i* (11990R-2^†^), *UAS-dlg1-i* (1725R-1), and *UAS-FasIII-i* (5803R-1) from National Institute of Genetics, Japan; and *Hsp70-GAL4* (1799), *Cg-GAL4* (7011) [56], *HmlΔ-GAL4* (30141), *ptc-GAL4* (2017), *AB1-GAL4* (1824), *Act5C-GAL4* (3954), *UAS-Dcr2* (24650), *UAS-Ser.mg5603* (5815), *UAS-GFP* (4775), *LanB2^MI03747^* (37366), *hop^Tum^* (8492), *spag^K12101^* (12200), *UAS-LanA-i* (28071), *UAS-trol-i* (29440^†^), *UAS-mys-i* (33642, 27735), *UAS-cora-i* (28993), *UAS-dlg1-i* (33620), *UAS-scrib-i* (29552), *UAS-shg-i* (32904), and *UAS-arm-i* (35004) from the Bloomington Stock Center. The following stocks were obtained from private collections: *Eater-GFP*(X) from R. A. Schulz [57]; *FB-GAL4* from R. P. Kühnlein [58]; and *UAS-dome^ΔCYT^*, *vkg^G454^*, and *UAS-Mmp2* from T. Xu [18]. The RNAi constructs marked with † proved stronger in knockdown experiments than others and were used for further analysis including imaging.

### Culture conditions and heat shock induction

All flies were maintained at 25°C unless otherwise indicated on standard cornmeal and agar media. For RNAi knockdown using *Hsp70-GAL4*, a single heat pulse of 30 min at 37°C was applied at 24, 48, 72, or 96 h after egg laying (AEL). After counting larval black nodules, 48 h and 96 h time points were chosen as the two optimal conditions for later experiments as 24 h yielded a lower rate of melanotic mass induction and 72 h resulted in a higher rate of lethality. For imaging the BM, a single heat-shock pulse was applied at 48 h AEL for *Hsp70>vkg-i*, and double pulses were applied at 48 h and 96 h AEL for *Hsp70>LanB2-i^104013^;+/LanB2^MI03747^* for stronger knockdown.

### Counting melanotic masses

Larval density and stage were tightly controlled during culture due to the fact that melanotic mass formation was affected by overcrowding growth conditions. Ten virgin females of *GAL4* strains were crossed to 5–7 males carrying different *UAS-RNA-i* strains. After 2 days, eggs were collected for 6 h (approximately 40–60 eggs). Melanotic masses were counted at the wandering third instar stage by rotating the larvae under the dissection microscope. The percentages of larvae containing at least one melanotic mass in their bodies were determined after counting 3 or more vials (n≥150) for each genotype or developmental time point.

### Immunostaining, antibodies, and imaging

Wandering third instar larvae were dissected on a silicone pad in phosphate-buffered saline (PBS) using a pair of forceps. Larval organs were transferred immediately to 4% paraformaldehyde (PFA) and fixed for 15–30 min at room temperature. Samples were washed four times in PBS plus 0.1% Tween-20 (PBST) and incubated in a blocking solution of PBST plus 5% normal goat serum for 1 h (PBST-NGS). Samples were then incubated with primary antibodies in PBST-NGS overnight at 4°C and then washed four times with PBST-NGS. Samples were then incubated with secondary antibodies alone or together with phalloidin-FITC in PBST-NGS for 2 h at room temperature. After four washes, samples were mounted in Vectorshield plus DAPI (Vector Laboratories, Inc., Burlingame, CA) and were subjected to fluorescent microscopy (Olympus BX40) or confocal microscopy (Zeiss LSM 510 META). For junction staining of the salivary gland, PBS plus 0.3% Triton X-100 was used as the buffer. The following antibodies and reagents were used: mouse anti-collagen IV [59] (Col IV) (6G7, 1∶100), rabbit anti-LanB1 (1∶500; Abcam), rabbit anti-LanB2 (1∶500; Abcam), rabbit anti-Trol [60] (1∶2000), rabbit anti-Ndg [61] (1∶2000), mouse anti-Hemes [62] (H2) (1∶500), mouse anti-L1 [63] (1∶500), mouse anti-Mys (1∶200; Developmental Studies Hybridoma Bank [DSHB]), mouse anti-Dlg 4F3 (1∶100; DSHB), mouse anti-Coracle C615.16 (1∶100; DSHB), anti-mouse IgG-Cy3 (1∶200; Jackson ImmunoResearch), phalloidin-FITC (1∶50 dilution of 400 µM stock; Sigma–Aldrich), and anti-rabbit and anti-mouse IgG conjugated to Alexa 488 or Alexa 546 (1∶200; Molecular Probes). For quantification of the BM fluorescence intensity, 400× magnified confocal images for the middle-marginal part of the salivary gland or region ‘6’ of the fat body were collected [11]. The fluorescence intensity was measured in 5–7 samples per genotype using the Image J software as described previously [26].

### Counting hemocytes

Wandering third instar larvae were washed in PBS and dried. A single larva was bled on a silicone pad in a 12-µl drop of PBS by ripping the epidermis with two fine forceps. The PBS/hemocyte drop was swirled gently using a micropipette tip and mounted on the Neubauer hemocytometer for counting. The total hemocytes were counted, and the lamellocytes were counted based on their characteristic morphology. For each genotype, at least 15 larvae were analyzed.

### Wounding

In situ wounding was performed as described previously [18] with minor modifications. Third instar larvae were immersed in water on a silicone pad. Under the GFP/dissection microscope, larvae were gently pinched at the salivary glands (with help of *AB1>GFP*) using a pair of forceps (Fine Science Tools, Cat. No. 11295-00). Larvae were transferred to fresh cornmeal-agar media and incubated at 25°C. Dead or melanized larvae that were identified within the first 3 h were removed. After 48 h of wounding, non-pupariated larvae were dissected and analyzed for melanotic mass formation on the salivary gland.

## Supporting Information

Figure S1 Analysis of melanotic mass formation in BM-deficient larvae and in extant mutants. (A) Immunostaining of black masses recovered from *Hsp70>vkg-i*, *Hsp70>LanB2-i*, and *Hsp70>mys-i* larvae. Lamellocytes were visualized following staining with anti-L1 antibody (red) and phalloidin-FITC (green). The nuclei were stained with DAPI (blue). (B) Induction of the antimicrobial peptide gene *Attacin-A* was analyzed by real-time PCR in *Hsp70>vkg-i* larvae. *Rp49* was used as a loading control. Error bars represent standard deviations (SD). (C) Confocal images of the BM of the larval fat bodies of the indicated genotypes. The control represents *Cg-GAL4* only. Collagen IV and laminin were stained with anti-Col IV (green) and anti-LanB1 (red), respectively. The nuclei were stained with DAPI (blue). (D) Immunostaining of the larval fat bodies or salivary glands of the indicated genotypes. Lamellocytes were stained with anti-L1 antibodies (red) and phalloidin-FITC (green). The nuclei were stained with DAPI (blue). Scale bar: 50 µm (A, C) and 100 µm (D).
(TIF)

Figure S2 Analysis of the activation state of hemocytes in the BM-deficient larvae. (A, B) Numbers of crystal cells were counted in larvae of the indicated genotypes. *Serrate* (*Ser*) was used as a positive control [64]. Error bars represent SEM. (C) Sessile hemocytes were analyzed using the plasmatocyte-specific *Eater-GFP*. Arrowheads indicate segmentally arranged sessile hemocytes. (D) Confocal images of circulating hemocytes after visualization of laminin following staining with anti-LanB1 antibodies (red), collagen IV with Vkg-GFP (green), F-actin following staining with phalloidin-FITC (green), and nuclei following staining with DAPI (blue). Scale bar: 500 µm (A, C), and 10 µm (D).
(TIF)

Figure S3 Nidogen and Perlecan are not necessary for blocking melanotic mass formation against self-tissue. (A–C) Confocal images of fat-body BMs after visualization of collagen IV using Vkg-GFP (green), laminin using anti-LanB1 antibodies (red), and nuclei using DAPI (blue). The control is *vkg^G454^*/+. (D) Quantitation of the fluorescence intensities in (A–C). Error bars represent SEM. **p<0.01 and ***p<0.001 by Student's *t*-test. (E) Confocal images of salivary gland and fat body BMs from Oregon R and *Act5C>trol-i, Dicer2* larvae. Perlecan and cell nuclei were stained with anti-Trol antibodies (purple) and DAPI (blue), respectively. (F) Confocal images of salivary gland and fat body BMs from Oregon R and *FB>vkg-i* larvae. Nidogen and cell nuclei were stained with anti-Nidogen antibodies (cyan) and DAPI (blue), respectively. BM Nidogen disappeared in salivary glands and fat bodies of *FB>vkg-i* larvae. It should be noted, however, that in embryos and wing discs BM Nidogen has been shown to be unaffected by collagen IV knockdown [26]. Scale bars: 50 µm.
(TIF)

Figure S4 Mmp2 overexpression disrupts the BM but does not induce melanotic mass formation in the salivary gland. (A, B) Confocal images of the salivary-gland BM of *GAL4*-only (A) and *ptc>Mmp2* (B) larvae after staining for collagen IV with anti-Col IV antibodies (green), laminin with anti-LanB2 antibodies (red), and nuclei with DAPI (blue). (C, D) Hemocyte attachment (anti-Hem in red) to the salivary glands of (A, B) was analyzed. Nuclei were stained with DAPI (blue). Scale bar: 50 µm (A, B) and 100 µm (C, D).
(TIF)

Figure S5 Knockdown phenotypes for an additional *mys-i* construct. (A) Confirmation of *mys* knockdown in fat bodies after immunostaining for Mys (anti-Mys in red). The control was *FB-GAL*4 only. (B) In the salivary glands of *ptc>mys-i^27735^*, apicobasal cell polarity was similarly disrupted as in *AB1>mys-i^33642^* larvae (see Figure 4I, J). Cora and Dlg were stained with anti-Cora (red) and anti-Dlg (red) antibodies, respectively. F-actin and nuclei were stained with phalloidin-FITC (green) and DAPI (blue), respectively. (C) The BM remained intact despite the obvious defects in cell polarity and cell-cell adhesion, and these characteristics were not different from those in *AB1>mys-i^33642^* larvae (see Figure 4K). BM collagen IV and BM laminin were visualized by Vkg-GFP (green) and anti-LanB2 antibodies (red), respectively. Scale bar: 50 µm.
(TIF)

Table S1 Melanotic mass formation after RNAi knockdown of various BM components.
(PDF)

Table S2 Examination of melanotic mass-associated genes.
(PDF)

Text S1 Supplementary methods.
(DOCX)

## References

[1] Takeuchi O, Akira S (2010) Pattern Recognition Receptors and Inflammation. Cell 140: 805–820. 20303872 10.1016/j.cell.2010.01.022

[2] Medzhitov R, Preston-Hurlburt P, Janeway CA Jr (1997) A human homologue of the Drosophila toll protein signals activation of adaptive immunity. Nature 388: 394–397. 9237759 10.1038/41131

[3] Marshak-Rothstein A, Rifkin IR (2007) Immunologically active autoantigens: the role of toll-like receptors in the development of chronic inflammatory disease. Annu Rev Immunol 25: 419–441. 17378763 10.1146/annurev.immunol.22.012703.104514

[4] Watson KL, Johnson TK, Denell RE (1991) Lethal(1) aberrant immune response mutations leading to melanotic tumor formation in Drosophila melanogaster. Developmental Genetics 12: 173–187. 1907895 10.1002/dvg.1020120302

[5] Qiu P, Pan PC, Govind S (1998) A role for the Drosophila Toll/Cactus pathway in larval hematopoiesis. Development 125: 1909–1920. 9550723 10.1242/dev.125.10.1909

[6] Minakhina S, Steward R (2006) Melanotic mutants in Drosophila: Pathways and phenotypes. Genetics 174: 253–263. 16816412 10.1534/genetics.106.061978 PMC1569781

[7] Rizki RM, Rizki TM (1984) Selective destruction of a host blood cell type by a parasitoid wasp. Proceedings of the National Academy of Sciences of the United States of America 81: 6154–6158. 6435126 10.1073/pnas.81.19.6154 PMC391878

[8] Meister M (2004) Blood cells of Drosophila: Cell lineages and role in host defence. Current Opinion in Immunology 16: 10–15. 14734104 10.1016/j.coi.2003.11.002

[9] Rizki RM, Rizki TM (1974) Basement membrane abnormalities in melanotic tumor formation of Drosophila. Experientia 30: 543–546. 4208981 10.1007/BF01926343

[10] Rizki TM, Rizki RM (1980) Developmental analysis of a temperature-sensitive melanotic tumor mutant in Drosophila melanogaster. Wilhelm Roux's Archives of Developmental Biology 189: 197–206. 10.1007/BF00868678 28305175

[11] Rizki RM, Rizki TM (1980) Hemocyte responses to implanted tissues in Drosophila melanogaster larvae. Wilhelm Roux's Archives of Developmental Biology 189: 207–213. 10.1007/BF00868679 28305176

[12] Rodriguez A, Zhou Z, Tang ML, Meller S, Chen J, et al (1996) Identification of immune system and responses genes, and novel mutations causing melanotic tumor formation in Drosophila melanogaster. Genetics 143: 929–940. 8725239 10.1093/genetics/143.2.929 PMC1207349

[13] Henchcliffe C, Garcia-Alonso L, Tang J, Goodman CS (1993) Genetic analysis of laminin A reveals diverse functions during morphogenesis in Drosophila. Development 118: 325–337. 8223265 10.1242/dev.118.2.325

[14] Urbano JM, Torgler CN, Molnar C, Tepass U, López-Varea A, et al (2009) Drosophila laminins act as key regulators of basement membrane assembly and morphogenesis. Development 136: 4165–4176. 19906841 10.1242/dev.044263 PMC2781052

[15] Wolfstetter G, Holz A (2012) The role of LamininB2 (LanB2) during mesoderm differentiation in Drosophila. Cellular and Molecular Life Sciences 69: 267–282. 21387145 10.1007/s00018-011-0652-3 PMC11114671

[16] Martin D, Zusman S, Li X, Williams EL, Khare N, et al (1999) wing blister, a new Drosophila laminin alpha chain required for cell adhesion and migration during embryonic and imaginal development. Journal of Cell Biology 145: 191–201. 10189378 10.1083/jcb.145.1.191 PMC2148222

[17] Babcock DT, Brock AR, Fish GS, Wang Y, Perrin L, et al (2008) Circulating blood cells function as a surveillance system for damaged tissue in Drosophila larvae. Proceedings of the National Academy of Sciences of the United States of America 105: 10017–10022. 18632567 10.1073/pnas.0709951105 PMC2474562

[18] Pastor-Pareja JC, Ming W, Tian X (2008) An innate immune response of blood cells to tumors and tissue damage in Drosophila. DMM Disease Models and Mechanisms 1: 144–154. 19048077 10.1242/dmm.000950 PMC2562178

[19] Paddibhatla I, Lee MJ, Kalamarz ME, Ferrarese R, Govind S (2010) Role for sumoylation in systemic inflammation and immune homeostasis in Drosophila larvae. PLoS Pathogens 6: e1001234. 21203476 10.1371/journal.ppat.1001234 PMC3009591

[20] Sasaki T, Fässler R, Hohenester E (2004) Laminin: The crux of basement membrane assembly. Journal of Cell Biology 164: 959–963. 15037599 10.1083/jcb.200401058 PMC2172061

[21] Yurchenco PD (2011) Basement membranes: Cell scaffoldings and signaling platforms. Cold Spring Harbor Perspectives in Biology 3: 1–27. 10.1101/cshperspect.a004911 PMC3039528 21421915

[22] Colognato H, Yurchenco PD (2000) Form and function: The laminin family of heterotrimers. Developmental Dynamics 218: 213–234. 10842354 10.1002/(SICI)1097-0177(200006)218:2<213::AID-DVDY1>3.0.CO;2-R

[23] Natzle JE, Monson JM, McCarthy BJ (1982) Cytogenetic location and expression of collagen-like genes in Drosophila. Nature 296: 368–371. 7063036 10.1038/296368a0

[24] Yasothornsrikul S, Davis WJ, Cramer G, Kimbrell DA, Dearolf CR (1997) viking: identification and characterization of a second type IV collagen in Drosophila. Gene 198: 17–25. 9370260 10.1016/s0378-1119(97)00274-6

[25] Montell DJ, Goodman CS (1988) Drosophila substrate adhesion molecule: sequence of laminin B1 chain reveals domains of homology with mouse. Cell 53: 463–473. 3365769 10.1016/0092-8674(88)90166-3

[26] Pastor-Pareja J, Xu T (2011) Shaping Cells and Organs in Drosophila by Opposing Roles of Fat Body-Secreted Collagen IV and Perlecan. Developmental Cell 21: 245–256. 21839919 10.1016/j.devcel.2011.06.026 PMC4153364

[27] Kusche-Gullberg M, Garrison K, MacKrell AJ, Fessler LI, Fessler JH (1992) Laminin A chain: expression during Drosophila development and genomic sequence. The EMBO Journal 11: 4519–4527. 1425586 10.1002/j.1460-2075.1992.tb05553.x PMC557027

[28] Avet-Rochex A, Boyer K, Polesello C, Gobert V, Osman D, et al (2010) An in vivo RNA interference screen identifies gene networks controlling Drosophila melanogaster blood cell homeostasis. BMC Developmental Biology 10: 65. 20540764 10.1186/1471-213X-10-65 PMC2891661

[29] Harrison DA, Binari R, Nahreini TS, Gilman M, Perrimon N (1995) Activation of a Drosophila Janus kinase (JAK) causes hematopoietic neoplasia and developmental defects. The EMBO Journal 14: 2857–2865. 7796812 10.1002/j.1460-2075.1995.tb07285.x PMC398404

[30] Luo H, Rose P, Barber D, Hanratty WP, Lee S, et al (1997) Mutation in the Jak kinase JH2 domain hyperactivates Drosophila and mammalian Jak-Stat pathways. Molecular and Cellular Biology 17: 1562–1571. 9032284 10.1128/mcb.17.3.1562 PMC231882

[31] Evans CJ, Hartenstein V, Banerjee U (2003) Thicker than blood: Conserved mechanisms in Drosophila and vertebrate hematopoiesis. Developmental Cell 5: 673–690. 14602069 10.1016/s1534-5807(03)00335-6

[32] Brown S, Hu N, Castelli-Gair Hombria J (2001) Identification of the first invertebrate interleukin JAK/STAT receptor, the Drosophila gene domeless. Current Biology 11: 1700–1705. 11696329 10.1016/s0960-9822(01)00524-3

[33] Luo H, Rose P, Roberts T, Dearolf C (2002) The Hopscotch Jak kinase requires the Raf pathway to promote blood cell activation and differentiation in Drosophila. Molecular Genetics and Genomics 267: 57–63. 11919715 10.1007/s00438-001-0632-7

[34] Huang L, Ohsako S, Tanda S (2005) The lesswright mutation activates Rel-related proteins, leading to overproduction of larval hemocytes in Drosophila melanogaster. Developmental Biology 280: 407–420. 15882582 10.1016/j.ydbio.2005.02.006

[35] Sorrentino RP, Tokusumi T, Schulz RA (2007) The Friend of GATA protein U-shaped functions as a hematopoietic tumor suppressor in Drosophila. Developmental Biology 311: 311–323. 17936744 10.1016/j.ydbio.2007.08.011

[36] Markovic MP, Kylsten P, Dushay MS (2009) Drosophila lamin mutations cause melanotic mass formation and lamellocyte differentiation. Molecular Immunology 46: 3245–3250. 19716177 10.1016/j.molimm.2009.08.003

[37] Rizki TM, Rizki RM (1992) Lamellocyte differentiation in Drosophila larvae parasitized by Leptopilina. Developmental and Comparative Immunology 16: 103–110. 1499832 10.1016/0145-305x(92)90011-z

[38] Tokusumi T, Sorrentino RP, Russell M, Ferrarese R, Govind S, et al (2009) Characterization of a lamellocyte transcriptional enhancer located within the misshapen gene of Drosophila melanogaster. PLoS ONE 4: e6429. 19641625 10.1371/journal.pone.0006429 PMC2713827

[39] Márkus R, Laurinyecz B, Kurucz É, Honti V, Bajusz I, et al (2009) Sessile hemocytes as a hematopoietic compartment in Drosophila melanogaster. Proceedings of the National Academy of Sciences of the United States of America 106: 4805–4809. 19261847 10.1073/pnas.0801766106 PMC2660760

[40] Sinenko SA, Mathey-Prevot B (2004) Increased expression of Drosophila tetraspanin, Tsp68C, suppresses the abnormal proliferation of ytr-deficient and Ras/Raf-activated hemocytes. Oncogene 23: 9120–9128. 15480416 10.1038/sj.onc.1208156

[41] Woods DF, Wu JOW, Bryant PJ (1997) Localization of proteins to the apico-lateral junctions of Drosophila epithelia. Developmental Genetics 20: 111–118. 9144922 10.1002/(SICI)1520-6408(1997)20:2<111::AID-DVG4>3.0.CO;2-A

[42] Munro S, Freeman M (2000) The Notch signalling regulator Fringe acts in the Golgi apparatus and requires the glycosyltransferase signature motif DXD. Current Biology 10: 813–820. 10899003 10.1016/s0960-9822(00)00578-9

[43] Warburg A, Shtern A, Cohen N, Dahan N (2007) Laminin and a Plasmodium ookinete surface protein inhibit melanotic encapsulation of Sephadex beads in the hemocoel of mosquitoes. Microbes and Infection 9: 192–199. 17224290 10.1016/j.micinf.2006.11.006

[44] Salt G (1970) The cellular defence reactions of insects: Cambridge University Press.

[45] Nacer A, Walker K, Hurd H (2008) Localisation of laminin within Plasmodium berghei oocysts and the midgut epithelial cells of Anopheles stephensi. Parasites & Vectors 1: 33. 18808667 10.1186/1756-3305-1-33 PMC2556657

[46] Goodnow CC (2007) Multistep Pathogenesis of Autoimmune Disease. Cell 130: 25–35. 17632054 10.1016/j.cell.2007.06.033

[47] Rasmussen JP, Reddy SS, Priess JR (2012) Laminin is required to orient epithelial polarity in the C. elegans pharynx. Development 139: 2050–2060. 22535412 10.1242/dev.078360 PMC3347693

[48] Pagliarini RA, Xu T (2003) A Genetic Screen in Drosophila for Metastatic Behavior. Science 302: 1227–1231. 14551319 10.1126/science.1088474

[49] Bilder D (2004) Epithelial polarity and proliferation control: Links from the Drosophila neoplastictumor suppressors. Genes and Development 18: 1909–1925. 15314019 10.1101/gad.1211604

[50] Irving-Rodgers HF, Ziolkowski AF, Parish CR, Sado Y, Ninomiya Y, et al (2008) Molecular composition of the peri-islet basement membrane in NOD mice: A barrier against destructive insulitis. Diabetologia 51: 1680–1688. 18633594 10.1007/s00125-008-1085-x PMC2516190

[51] Korpos E, Kadri N, Kappelhoff R, Wegner J, Overall CM, et al (2013) The peri-islet basement membrane, a barrier to infiltrating leukocytes in type 1 diabetes in mouse and human. Diabetes 62: 531–542. 23139348 10.2337/db12-0432 PMC3554379

[52] Laine M, Virtanen I, Salo T, Konttinen YT (2004) Segment-specific but pathologic laminin isoform profiles in human labial salivary glands of patients with Sjögren's syndrome. Arthritis and Rheumatism 50: 3968–3973. 15593200 10.1002/art.20730

[53] Laine M, Virtanen I, Porola P, Rotar Z, Rozman B, et al (2008) Acinar epithelial cell laminin-receptors in labial salivary glands in Sjögren's syndrome. Clinical and Experimental Rheumatology 26: 807–813. 19032812

[54] Karre K, Ljunggren HG, Piontek G, Kiessling R (1986) Selective rejection of H-2-deficient lymphoma variants suggests alternative immune defence strategy. Nature 319: 675–678. 3951539 10.1038/319675a0

[55] Vivier E, Raulet DH, Moretta A, Caligiuri MA, Zitvogel L, et al (2011) Innate or adaptive immunity? The example of natural killer cells. Science 331: 44–49. 21212348 10.1126/science.1198687 PMC3089969

[56] Asha H, Nagy I, Kovacs G, Stetson D, Ando I, et al (2003) Analysis of ras-induced overproliferation in Drosophila hemocytes. Genetics 163: 203–215. 12586708 10.1093/genetics/163.1.203 PMC1462399

[57] Tokusumi T, Shoue DA, Tokusumi Y, Stoller JR, Schulz RA (2009) New hemocyte-specific enhancer-reporter transgenes for the analysis of hematopoiesis in Drosophila. Genesis 47: 771–774. 19830816 10.1002/dvg.20561

[58] Gronke S, Beller M, Fellert S, Ramakrishnan H, Jackle H, et al (2003) Control of fat storage by a Drosophila PAT domain protein. Current Biology 13: 603–606. 12676093 10.1016/s0960-9822(03)00175-1

[59] Murray M, Fessler L, Palka J (1995) Changing Distributions of Extracellular Matrix Components during Early Wing Morphogenesis in Drosophila. Developmental biology 168: 150–165. 7883070 10.1006/dbio.1995.1068

[60] Friedrich MVK, Schneider M, Timpl R, Baumgartner S (2000) Perlecan domain V of Drosophila melanogaster. Sequence, recombinant analysis and tissue expression. European Journal of Biochemistry 267: 3149–3159. 10824099 10.1046/j.1432-1327.2000.01337.x

[61] Wolfstetter G, Shirinian M, Stute C, Grabbe C, Hummel T, et al (2009) Fusion of circular and longitudinal muscles in Drosophila is independent of the endoderm but further visceral muscle differentiation requires a close contact between mesoderm and endoderm. Mechanisms of Development 126: 721–736. 19463947 10.1016/j.mod.2009.05.001

[62] Kurucz E, Zettervall CJ, Sinka R, Vilmos P, Pivarcsi A, et al (2003) Hemese, a hemocyte-specific transmembrane protein, affects the cellular immune response in Drosophila. Proceedings of the National Academy of Sciences of the United States of America 100: 2622–2627. 12598653 10.1073/pnas.0436940100 PMC151390

[63] Kurucz E, Váczi B, Márkus R, Laurinyecz B, Vilmos P, et al (2007) Definition of Drosophila hemocyte subsets by cell-type specific antigens. Acta Biologica Hungarica 58: 95–111. 18297797 10.1556/ABiol.58.2007.Suppl.8

[64] Duvic B, Hoffmann JA, Meister M, Royet J (2002) Notch signaling controls lineage specification during Drosophila larval hematopoiesis. Current Biology 12: 1923–1927. 12445385 10.1016/s0960-9822(02)01297-6

