# Supplementary figures and images for: Basement Membrane and Cell Integrity of Self-Tissues in Maintaining Drosophila Immunological Tolerance
Source: PLoS Genet. 2014 Oct 16;10(10):e1004683. doi: 10.1371/journal.pgen.1004683 (PMC4199487; doi:10.1371/journal.pgen.1004683)

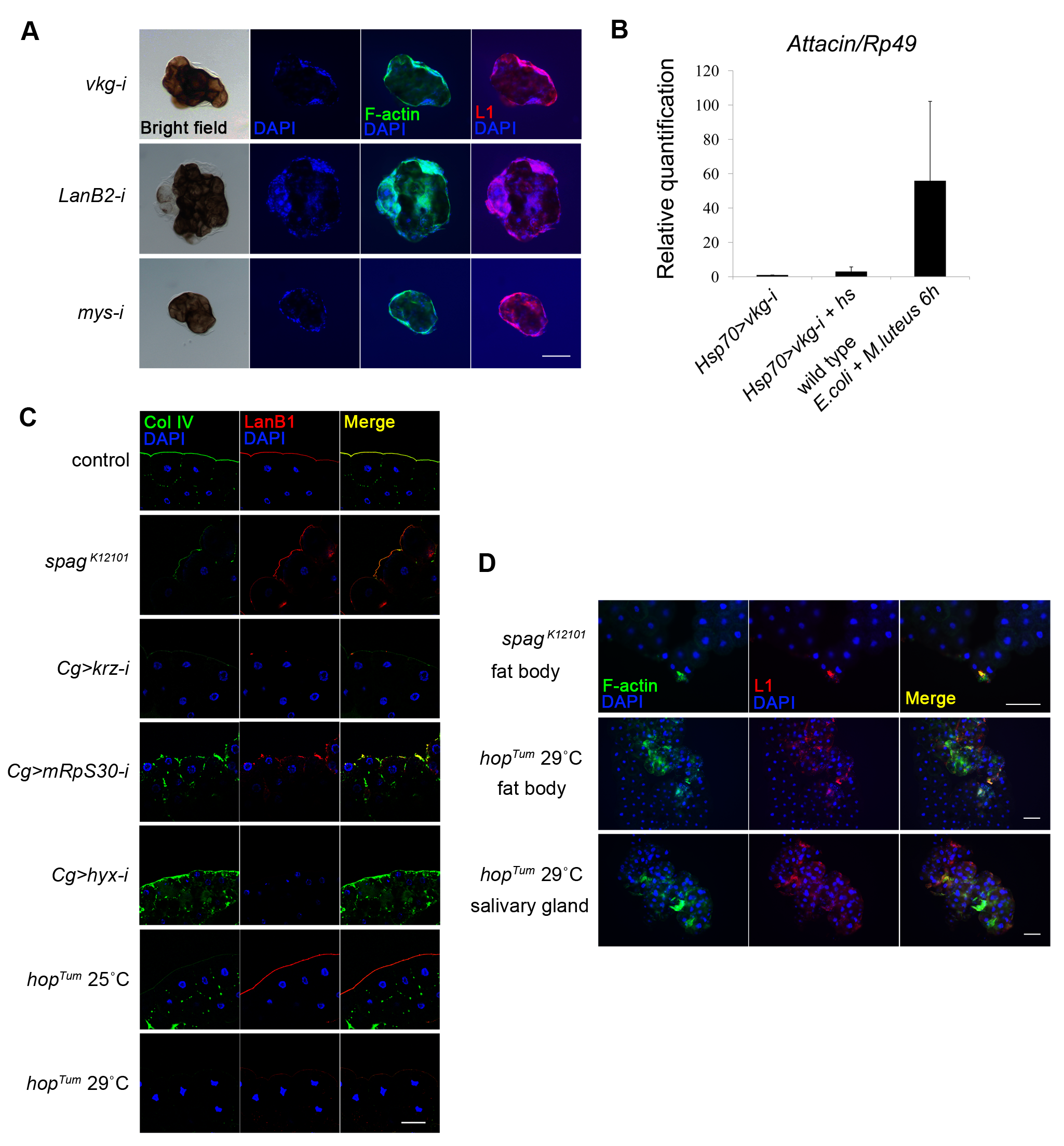

Supplement: Figure S1 — Analysis of melanotic mass formation in BM-deficient larvae and in extant mutants. (A) Immunostaining of black masses recovered from Hsp70>vkg-i, Hsp70>LanB2-i, and Hsp70>mys-i larvae. Lamellocytes were visualized following staining with anti-L1 antibody (red) and phalloidin-FITC (green). The nuclei were stained with DAPI (blue). (B) Induction of the antimicrobial peptide gene Attacin-A was analyzed by real-time PCR in Hsp70>vkg-i larvae. Rp49 was used as a loading control. Error bars represent standard deviations (SD). (C) Confocal images of the BM of the larval fat bodies of the indicated genotypes. The control represents Cg-GAL4 only. Collagen IV and laminin were stained with anti-Col IV (green) and anti-LanB1 (red), respectively. The nuclei were stained with DAPI (blue). (D) Immunostaining of the larval fat bodies or salivary glands of the indicated genotypes. Lamellocytes were stained with anti-L1 antibodies (red) and phalloidin-FITC (green). The nuclei were stained with DAPI (blue). Scale bar: 50 µm (A, C) and 100 µm (D). (TIF) [file pgen.1004683.s001.tif]

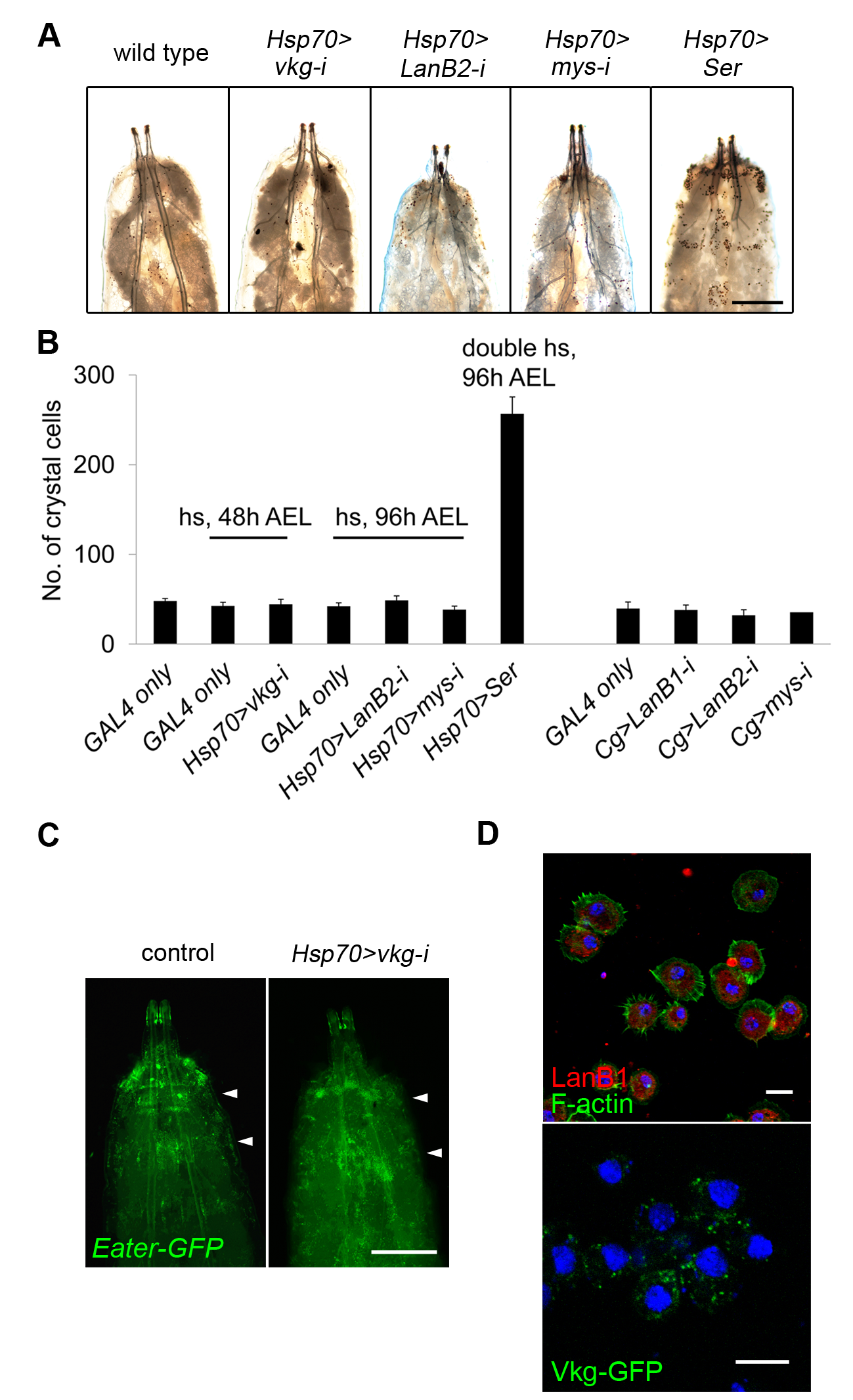

Supplement: Figure S2 — Analysis of the activation state of hemocytes in the BM-deficient larvae. (A, B) Numbers of crystal cells were counted in larvae of the indicated genotypes. Serrate (Ser) was used as a positive control [64]. Error bars represent SEM. (C) Sessile hemocytes were analyzed using the plasmatocyte-specific Eater-GFP. Arrowheads indicate segmentally arranged sessile hemocytes. (D) Confocal images of circulating hemocytes after visualization of laminin following staining with anti-LanB1 antibodies (red), collagen IV with Vkg-GFP (green), F-actin following staining with phalloidin-FITC (green), and nuclei following staining with DAPI (blue). Scale bar: 500 µm (A, C), and 10 µm (D). (TIF) [file pgen.1004683.s002.tif]

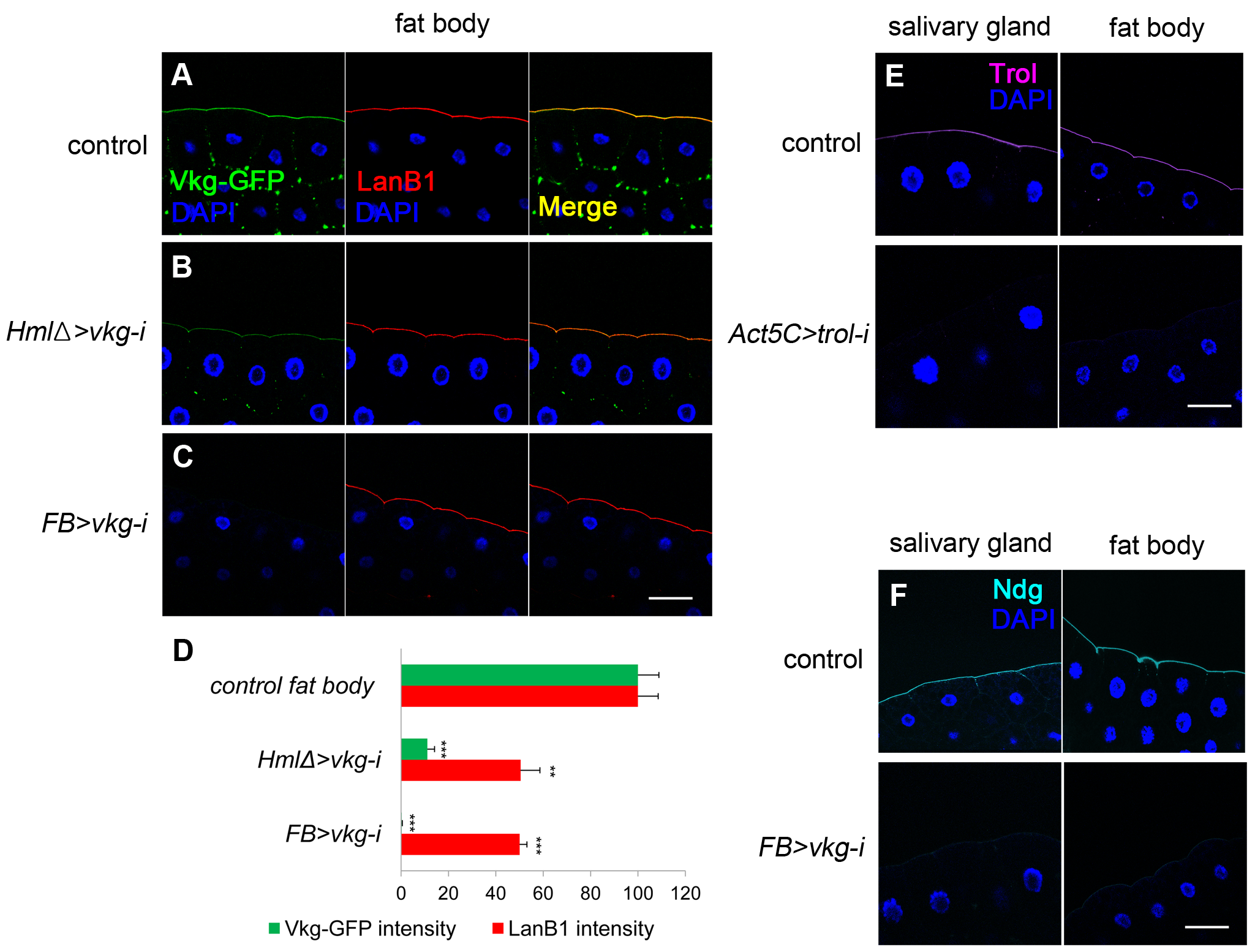

Supplement: Figure S3 — Nidogen and Perlecan are not necessary for blocking melanotic mass formation against self-tissue. (A–C) Confocal images of fat-body BMs after visualization of collagen IV using Vkg-GFP (green), laminin using anti-LanB1 antibodies (red), and nuclei using DAPI (blue). The control is vkgG454/+. (D) Quantitation of the fluorescence intensities in (A–C). Error bars represent SEM. **p<0.01 and ***p<0.001 by Student's t-test. (E) Confocal images of salivary gland and fat body BMs from Oregon R and Act5C>trol-i, Dicer2 larvae. Perlecan and cell nuclei were stained with anti-Trol antibodies (purple) and DAPI (blue), respectively. (F) Confocal images of salivary gland and fat body BMs from Oregon R and FB>vkg-i larvae. Nidogen and cell nuclei were stained with anti-Nidogen antibodies (cyan) and DAPI (blue), respectively. BM Nidogen disappeared in salivary glands and fat bodies of FB>vkg-i larvae. It should be noted, however, that in embryos and wing discs BM Nidogen has been shown to be unaffected by collagen IV knockdown [26]. Scale bars: 50 µm. (TIF) [file pgen.1004683.s003.tif]

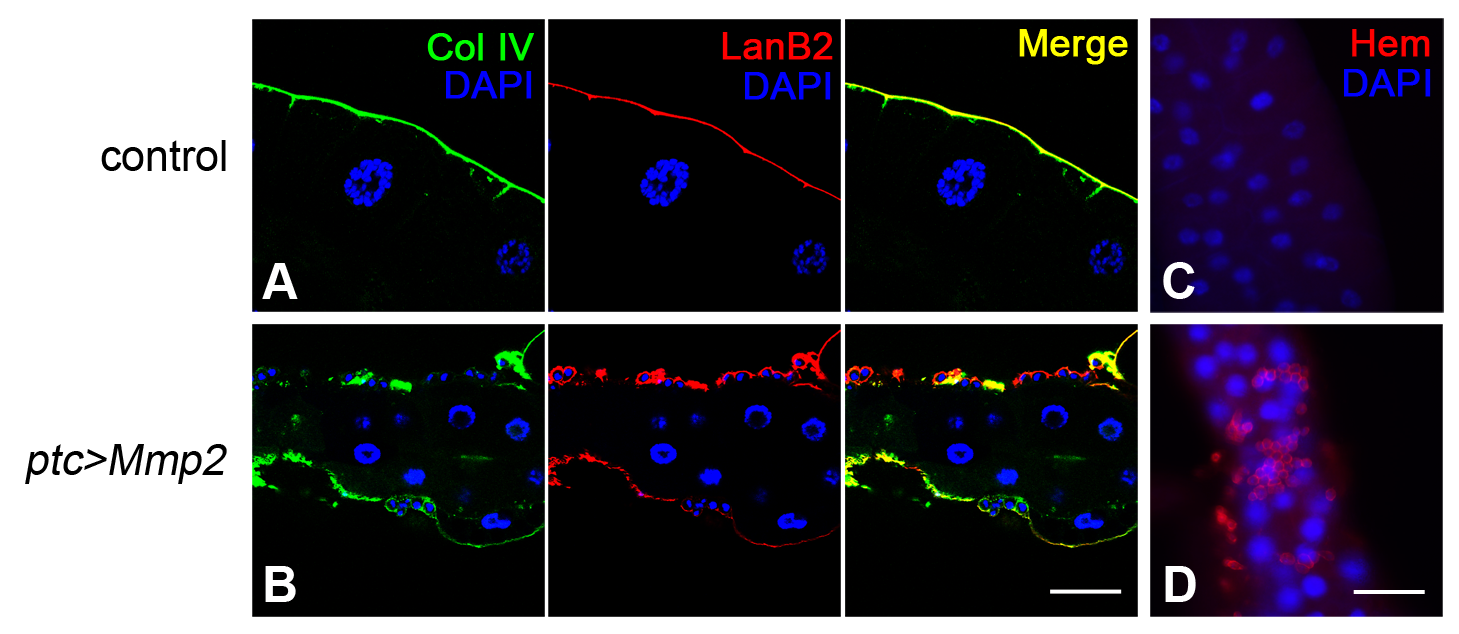

Supplement: Figure S4 — Mmp2 overexpression disrupts the BM but does not induce melanotic mass formation in the salivary gland. (A, B) Confocal images of the salivary-gland BM of GAL4-only (A) and ptc>Mmp2 (B) larvae after staining for collagen IV with anti-Col IV antibodies (green), laminin with anti-LanB2 antibodies (red), and nuclei with DAPI (blue). (C, D) Hemocyte attachment (anti-Hem in red) to the salivary glands of (A, B) was analyzed. Nuclei were stained with DAPI (blue). Scale bar: 50 µm (A, B) and 100 µm (C, D). (TIF) [file pgen.1004683.s004.tif]

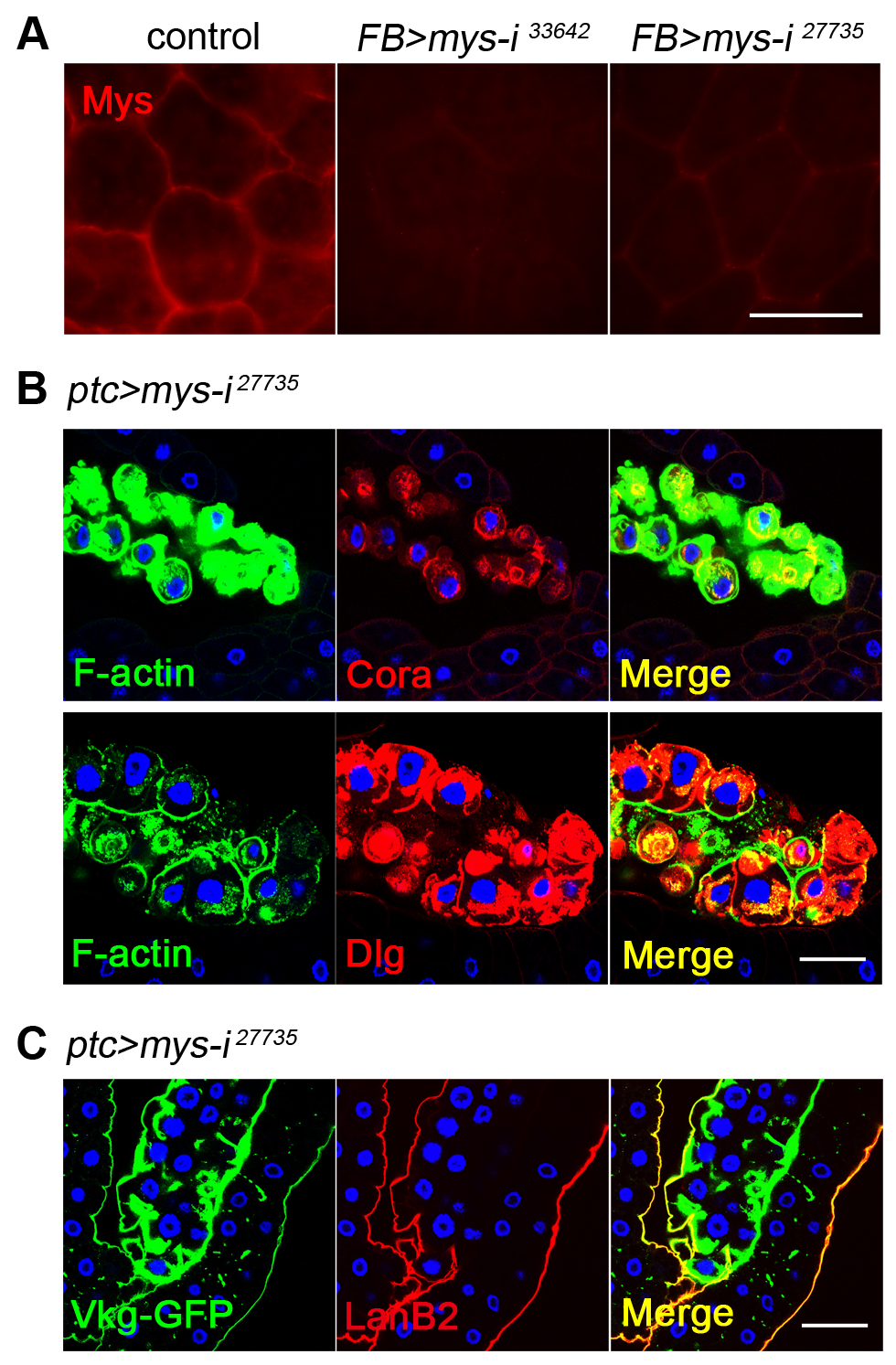

Supplement: Figure S5 — Knockdown phenotypes for an additional mys-i construct. (A) Confirmation of mys knockdown in fat bodies after immunostaining for Mys (anti-Mys in red). The control was FB-GAL4 only. (B) In the salivary glands of ptc>mys-i27735, apicobasal cell polarity was similarly disrupted as in AB1>mys-i33642 larvae (see Figure 4I, J). Cora and Dlg were stained with anti-Cora (red) and anti-Dlg (red) antibodies, respectively. F-actin and nuclei were stained with phalloidin-FITC (green) and DAPI (blue), respectively. (C) The BM remained intact despite the obvious defects in cell polarity and cell-cell adhesion, and these characteristics were not different from those in AB1>mys-i33642 larvae (see Figure 4K). BM collagen IV and BM laminin were visualized by Vkg-GFP (green) and anti-LanB2 antibodies (red), respectively. Scale bar: 50 µm. (TIF) [file pgen.1004683.s005.tif]
