# Supplementary material for: Basement Membrane and Cell Integrity of Self-Tissues in Maintaining Drosophila Immunological Tolerance
Source: PLoS Genet. 2014 Oct 16;10(10):e1004683. doi: 10.1371/journal.pgen.1004683 (PMC4199487; doi:10.1371/journal.pgen.1004683)
Supplement: Table S1 — Melanotic mass formation after RNAi knockdown of various BM components. (PDF) [file pgen.1004683.s006.pdf]

Table S1. Melanotic mass formation after RNAi knockdown of various BM components

Category	Gene	RNAi strain no.	*Act5C-GAL4*	*HmlΔ-GAL4*	*FB-GAL4*	*Cg-GAL4*	*Hsp70-GAL4*	
							48h AEL	96h AEL
control			-	-	-	-	-	2.63
BM	*vkg*	106812	E-L	-	-	2nd-L	73.90	36.25
		16858R-3	1st-L	-	-	3rd -L	5.40	-
	Cg25C	104536	E-L	-	-	2nd-L	75.90	27.00
		28369	E-L	-	-	2nd-L	28.00	-
	LanA	28071 (BL)	-	-	-	11.32	9.04	5.16
		18873	-	-	-	2.67	-	-
	wb	108020	P-L	-	-	-	15.30	24.60
	LanB1	23119	3rd-L *	-	-	38.39	-	-
		23121	E-L	-	-	41.81	-	-
	LanB2	42559	E-L	-	-	52.82	-	-
		42560	E-L	-	-	49.53	-	-
		104013	E-L	-	-	26.85	18.40	28.10
	trol	22642	-	-	-	-	-	-
		12497R-1	P-semi-L	-	-	-	-	-
	Ndg	13208	-	-	-	-	-	-
		12908R-3	P-semi-L	-	-	-	-	-
BM receptor-related	*mew* (αPS1)	44890	P-L	n.t.	n.t.	-	-	-
		1771R-1	-	n.t.	n.t.	-	-	-
	if (αPS2)	100770	-	n.t.	n.t.	-	-	-
		44885	E-L	n.t.	n.t.	-	-	-
	scb (αPS3)	4891	P-L	-	-	-	-	13.54
		100949	P-L	-	-	-	-	28.63
	ItgαPS4	16827R-2	-	n.t.	n.t.	-	-	-
		37172	-	n.t.	n.t.	-	-	-
	ItgαPS5	6646	-	n.t.	n.t.	-	-	-
		100120	-	n.t.	n.t.	-	-	-
	mys (βPS)	29619	E-L	-	-	44.62	L	49.64
		1560R-1	E-L	-	-	0.55	L	13.57
		33642 (BL)	E-L	-	-	3.05	L	33.96
	Itgβv	893	-	n.t.	n.t.	-	-	-
		1762R-1	-	n.t.	n.t.	-	-	-
	Dg	107029	-	-	-	-	20.10	16.80
	Dys	106401	-	-	-	-	L	25.53
		106578	-	-	-	-	L	25.00

The numbers of larvae containing melanotic masses are presented in percentages (%). For each experiment, n ≥ 150.
-: no melanotic mass was observed. n.t.: not tested. *: escapers had melanotic masses.
E-L: embryo lethal. 1st-L, 2nd-L, or 3rd-L: 1st, 2nd, or 3rd instar larva lethal, respectively. P-L: pupa lethal. P-semi-L: pupa semi-lethal. L: lethal (stage not determined).
Act5C-GAL4: ubiquitous expression with early onset [65].

HmlΔ-GAL4: larval hemocytes, lymph gland [40].
FB-GAL4: fat body, wing discs, central nerve system [28].
Cg-GAL4: embryonic and larval hemocytes, fat body [56].
Hsp70-GAL4: heat shock inducible [66].

References

65. Ito K, Awano W, Suzuki K, Hiromi Y, Yamamoto D (1997) The Drosophila mushroom body is a quadruple structure of clonal units each of which contains a virtually identical set of neurones and glial cells. Development 124: 761-771.
66. Brand AH, Perrimon N (1993) Targeted gene expression as a means of altering cell fates and generating dominant phenotypes. Development 118: 401-415.
