# Supplementary material for: Basement Membrane and Cell Integrity of Self-Tissues in Maintaining Drosophila Immunological Tolerance
Source: PLoS Genet. 2014 Oct 16;10(10):e1004683. doi: 10.1371/journal.pgen.1004683 (PMC4199487; doi:10.1371/journal.pgen.1004683)
Supplement: Table S2 — Examination of melanotic mass-associated genes. (PDF) [file pgen.1004683.s007.pdf]

Table S2. Examination of melanotic mass-associated genes

Gene name [ref]	CG no.	RNAi strain no.	*Act5C-GAL4*	*Cg-GAL4*	*FB-GAL4*	Information
abs [67]	CG14637	14637R-2	**-,** P-L	n.t.	n.t.	
Acn [68]	CG10473	10473R-1	-	n.t.	n.t.	
		10473R-3	-	n.t.	n.t.	
cact [19]	CG5848	5848R-1	-	n.t.	n.t.	BM of *ubc9* mutant larvae analyzed in [19]
		5848R-3	MM, P-L	n.t.	n.t.	
Cct2 [28]	CG18330	18330R-2	n.t.	-	-	
		18330R-3	n.t.	-	-	
CSN5 [28]	CG14884	14884R-1	-, MZ	n.t.	n.t.	MZ in 3rd instar larvae
Cul4 [28]	CG8711	44829	n.t.	MM	MM	FB, SG, and IDs absent (*Cg-GAL4*); a very rare MM (*FB-GAL4*)
		8711R-1	n.t.	-	-	
		8711R-2	n.t.	-	-	
Dcp-1 [6]	CG5370	5370R-1	-	n.t.	n.t.	
Dref [28]	CG5838	5838R-1	-, 2nd-L	n.t.	n.t.	
		5838R-4	-, MZ, P-L	n.t.	n.t.	MZ in 3rd instar larvae
gig [69]	CG6975	6975R-2	-	n.t.	n.t.	
		6975R-8	-	n.t.	n.t.	
heix [70]	CG5876	5876R-1	-, P-L	n.t.	n.t.	
		5876R-2	-	n.t.	n.t.	
Hsc70-4 [71]	CG4264	4264R-1	-, 2nd-L	n.t.	n.t.	
		4264R-2	-	n.t.	n.t.	
hyx [28]	CG11990	11990R-2	n.t.	MM	-	Figure S1
		11990R-3	n.t.	MM	-	
krz [6]	CG1487	103756	MM, P-L	MM	-	Figure S1
		41559	-	-	-	
		33880 (BL)	-	-	-	
		29523 (BL)	-	-	-	
		krz^{d1}	n.t.	n.t.	n.t.	A mutant allele; 1st-L
l(2)37Cd [68]	CG10563	10563R-2	-, MZ	n.t.	n.t.	MZ in some 3rd instar larvae
		10563R-3	-, MZ	n.t.	n.t.	
l(2)37Ce [68]	CG17347	17347R-1	-	n.t.	n.t.	
		17347R-2	-	n.t.	n.t.	
l(3)mbn [6]	CG12755	12755R-1	-	n.t.	n.t.	
		12755R-2	-	n.t.	n.t.	
Lim3 [72]	CG10699	10699R-4	-	n.t.	n.t.	
		10699R-5	-	n.t.	n.t.	
mib2 [68]	CG17492	17492R-1	-	n.t.	n.t.	
		17492R-3	-	n.t.	n.t.	
morgue [73]	CG15437	15437R-1	-	n.t.	n.t.	

		15437R-2	-, MZ	n.t.	n.t.	MZ in pupae
mrn [74]	CG7764	7764R-2	-	n.t.	n.t.	
mRpS30 [28]	CG8470	8470R-4	n.t.	MM	-	Figure S1
		8470R-3	n.t.	MM	-	
Nc [6]	CG8091	8091R-1	-, MZ	n.t.	n.t.	MZ in pupae
pita [75]	CG3941	3941R-1	-	n.t.	n.t.	
		3941R-2	-, MZ	n.t.	n.t.	MZ in 3rd instar larvae
Pof [76]	CG3691	3691R-1	-	n.t.	n.t.	
		3691R-3	-	n.t.	n.t.	
pr-set7 [6]	CG3307	3307R-3	-, MZ	n.t.	n.t.	MZ in 3rd instar larvae
pUf68 [77]	CG12085	12085R-2	MM, 2nd-L	n.t.	n.t.	MM in LG of 2nd instar larvae
		12085R-4	MM, MZ, 2nd-L	n.t.	n.t.	MM in LG of 2nd instar larvae; MZ in trachea
rnh1 [78]	CG8729	8729R-1	-	n.t.	n.t.	
		8729R-2	-	n.t.	n.t.	
skpA [6]	CG16983	16983R-2	-, MZ	n.t.	n.t.	MZ in pupae
		16983R-3	-, 2nd-L	n.t.	n.t.	
spag [6]	CG13570	$spag^{k12101}$	n.t.	n.t.	n.t.	A mutant allele; Figure S1
Su(var)205 [6]	CG8409	8409R-2	-	n.t.	n.t.	
		8409R-4	-, MZ	n.t.	n.t.	MZ in some pupae
Top3α [79]	CG10123	10123R-1	-, 2nd-L	n.t.	n.t.	
Tsc1 [80]	CG6147	6147R-1	-, MZ	n.t.	n.t.	MZ in pupae
		6147R-2	-, P-L	n.t.	n.t.	
ush [35]	CG2762	2762R-4	n.t.	MM	-	Blood cell tumor [35]
		2762R-2	n.t.	MM	-	
Zfrp8 [6]	CG3260	3260R-2	-, P-L	n.t.	n.t.	
		3260R-6	-, P-L	n.t.	n.t.	

MM: melanotic masses ("melanotic nodules" [6]). MZ: melanization in larval cuticle, trachea, or pupal cuticle. -: no melanotic mass was observed. n.t.: not tested.
2nd-L or 3rd-L: 2nd or 3rd instar larva lethal, respectively. P-L: pupa lethal.
FB, SG, ID, and LG indicate fat body, salivary gland, imaginal disc, and lymph gland, respectively.

References

67. Schmucker D, Vorbruggen G, Yeghiayan P, Fan HQ, Jackle H, et al. (2000) The Drosophila gene abstrakt, required for visual system development, encodes a putative RNA helicase of the DEAD box protein family. Mechanisms of Development 91: 189-196.
68. Stathakis DG, Pentz ES, Freeman ME, Kullman J, Hankins GR, et al. (1995) The genetic and molecular organization of the Dopa decarboxylase gene cluster of Drosophila melanogaster. Genetics 141: 629-655.
69. Ito N, Rubin GM (1999) gigas, a Drosophila homolog of tuberous sclerosis gene product-2, regulates the cell cycle. Cell 96: 529-539.

70. Ashburner M, Misra S, Roote J, Lewis SE, Blazej R, et al. (1999) An exploration of the sequence of a 2.9-Mb region of the genome of Drosophila melanogaster. The Adh region. Genetics 153: 179-219.
71. Chang HC, Newmyer SL, Hull MJ, Ebersold M, Schmid SL, et al. (2002) Hsc70 is required for endocytosis and clathrin function in Drosophila. Journal of Cell Biology 159: 477-487.
72. Wright TRF (1996) The Wilhelmine E. Key 1992 Invitational lecture. Phenotypic analysis of the Dopa decarboxylase gene cluster mutants in Drosophila melanogaster. Journal of Heredity 87: 175-190.
73. Hays R, Wickline L, Cagan R (2002) Morgue mediates apoptosis in the Drosophila melanogaster retina by promoting degradation of DIAP1. Nature Cell Biology 4: 425-431.
74. Fregoso M, Laine JP, Aguilar-Fuentes J, Mocquet V, Reynaud E, et al. (2007) DNA repair and transcriptional deficiencies caused by mutations in the Drosophila p52 subunit of TFIIH generate developmental defects and chromosome fragility. Molecular and Cellular Biology 27: 3640-3650.
75. Laundrie B, Peterson JS, Baum JS, Chang JC, Fileppo D, et al. (2003) Germline cell death is inhibited by P-element insertions disrupting the dcp-1/pita nested gene pair in Drosophila. Genetics 165: 1881-1888.
76. Larsson J, Svensson MJ, Stenberg P, Makitalo M (2004) Painting of fourth in genus Drosophila suggests autosome-specific gene regulation. Proceedings of the National Academy of Sciences of the United States of America 101: 9728-9733.
77. Van Buskirk C, Schupbach T (2002) half pint regulates alternative splice site selection in Drosophila. Developmental Cell 2: 343-353.
78. Filippov V, Filippova M, Gill SS (2001) Drosophila RNase H1 is essential for development but not for proliferation. Molecular Genetics and Genomics 265: 771-777.
79. Plank JL, Hai Chu S, Reineke Pohlhaus J, Wilson-Sali T, Hsieh TS (2005) Drosophila melanogaster topoisomerase IIIalpha preferentially relaxes a positively or negatively supercoiled bubble substrate and is essential during development. Journal of Biological Chemistry 280: 3564-3573.
80. Potter CJ, Huang H, Xu T (2001) Drosophila Tsc1 functions with Tsc2 to antagonize insulin signaling in regulating cell growth, cell proliferation, and organ size. Cell 105: 357-368.
