# Supplementary material for: Basement Membrane and Cell Integrity of Self-Tissues in Maintaining Drosophila Immunological Tolerance
Source: PLoS Genet. 2014 Oct 16;10(10):e1004683. doi: 10.1371/journal.pgen.1004683 (PMC4199487; doi:10.1371/journal.pgen.1004683)
Supplement: Text S1 — Supplementary methods. (DOCX) [file pgen.1004683.s008.docx]

**Supplementary Methods**

**Fly strains**

*krz^d1^* was obtained from G. Roman [81]. RNAi transgenic strains with "R-" in their strain numbers were obtained from National Institute of Genetics, Japan. *spag^k12101^* and RNAi strains with "(BL)" were obtained from the Bloomington Stock Center. All other RNAi strains were obtained from the Vienna Drosophila Resource Center, Austria.

**Counting crystal cells**

Crystal cells were first visualized by heating third instar larvae at 60°C for 10 min, and the black dots in the last two posterior segments were counted at the dorsal side [82]. For each genotype, n ≥ 15.

**Imaging hemocytes**

Hemocytes were loaded on a silane-coated microscope slide (Muto Pure Chemicals, Cat. No. 5116-20F) and incubated for 30 min at room temperature in a humid chamber to allow cell attachment to the glass plate. Samples were fixed with 4% formaldehyde for 15 min, washed with PBS, permeabilized with 0.3% Triton X-100 in PBS, and washed three times with PBST-NGS. Samples were incubated with primary antibodies in PBST-NGS for 3 h at room temperature in a humid chamber. After washing three times in PBST-NGS, the samples were incubated with secondary antibodies in PBST-NGS for 1 h at room temperature. The remainder of the procedure was performed as described in the main text for immunostaining.

**Real-time PCR**

For *AttA* induction, 20–30 *Hsp70>vkg-i* wandering third instar larvae that had been treated with heat shock at 48 h AEL or left untreated were collected. For the positive control, 20 stage-matched larvae that were infected with *E. coli* and *M. luteus* were used. RNA was purified using the Trizol reagent (Invitrogen, Carlsbad, CA), and 2 µg of total RNA was used for cDNA synthesis with M-MLV reverse transcriptase (Promega, Madison, WI). Real-time PCR was performed using SYBR premix Ex Taq (Takara, #RR041A) and the ABI Prism 7300, and the results were analyzed using the comparative Ct method. *Rp49* mRNA was used as a control for normalization, and triplicate samples for each condition were averaged. The following primers were used: *AttA*, 5’-AGGTTCCTTAACCTCCAATC-3’ (forward) and 5’-CATGACCAGCATTGTTGTAG-3’ (backward) and *Rp49*, 5’-CAGTCGGATCGATATGCTAAGCTGT-3’ (forward) and 5’-TAACCGATGTTGGGCATCAGATACT-3’ (backward).

**References**

81. Roman G, He J, Davis RL (2000) kurtz, a novel nonvisual arrestin, is an essential neural gene in Drosophila. Genetics 155: 1281-1295.

82. Zettervall CJ, Anderl I, Williams MJ, Palmer R, Kurucz E, et al. (2004) A directed screen for genes involved in Drosophila blood cell activation. Proceedings of the National Academy of Sciences of the United States of America 101: 14192-14197.
